# Computational De Novo Design of Group II Introns Yields Highly Active Ribozymes

**DOI:** 10.1002/cbic.202500356

**Published:** 2025-06-30

**Authors:** Deni Szokoli, Noemi E. Nwosu, Lukas M. Glatt, Hannes Mutschler

**Affiliations:** ^1^ Department of Chemistry and Chemical Biology TU Dortmund University Otto‐Hahn‐Str. 4a 44227 Dortmund Germany

**Keywords:** nucleic acids, ribozymes, RNA, RNA design, splicing, synthetic biology

## Abstract

Group II introns (G2Is) are large self‐splicing ribozymes with emerging potential in biotechnological applications. Despite growing interest, their complexity has so far precluded efforts to design them from scratch. While computational approaches have enabled the design of small RNA catalysts, methods for engineering large ribozymes remain underdeveloped. Herein, the RNA inverse folding algorithm aRNAque is used to design G2Is from scratch, yielding three novel self‐splicing ribozymes with unusually stable structures. The designed intron Arq.I2 is revealed to be an unexpectedly proficient ribozyme in vitro, self‐splicing at a rate comparable to the fastest known G2Is. While most G2Is are believed to be inactive under intracellular conditions in the absence of maturase proteins, it is shown that Arq.I2 self‐splices in *Escherichia coli* cells. The results demonstrate that highly active variants of large and complex ribozymes can be designed de novo with relative ease using existing inverse folding algorithms, paving the way for the design of bespoke ribozymes derived from G2Is for the development of biotechnological tools.

## Graphical abstract

Group II Introns (G2Is) are large self‐splicing ribozymes with promising biotechnological applications. This study utilized RNA inverse folding to design three novel G2Is. The designed intron Arq.I2, an unexpectedly proficient ribozyme, self‐splices not only in vitro, but also in *Escherichia coli* cells. The results demonstrate that complex ribozyme variants can be designed de novo using existing inverse folding algorithms.

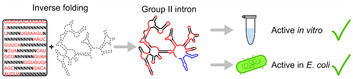

## 1 Introduction

RNA has recently found itself at the heart of a new technological and therapeutic revolution. The biological function of both coding and noncoding RNAs is inextricably linked to the folded structure the RNA adopts. Consequently, effective design of RNA‐based tools and therapeutics hinges on the ability to reliably and accurately produce RNAs that simultaneously fulfill certain structural and sequence‐based requirements. However, the free energy landscape for RNA folding is typically rugged^[^ ^1^, ^2^, ^3^, ^4^ ^]^ making engineering of functional RNA molecules with a defined 3D structure challenging. The energetic similarity of many RNA conformers can lead to drastic changes in the secondary and tertiary structure of RNA molecules due to even small sequence changes.^[^ ^5^, ^6^ ^]^ Numerous algorithms have been developed to solve the so‐called “inverse RNA folding problem”, which output an RNA sequence that is predicted to fold into a user‐specified secondary structure.^[^ ^7^, ^8^, ^9^, ^10^, ^11^, ^12^, ^13^, ^14^, ^15^, ^16^ ^]^ Only a handful of these algorithms have ever been experimentally validated for their ability to generate functional ribozymes, typically limited to relatively short ones such as the hammerhead, HDV, *glmS or hairpin* ribozymes.^[^ ^17^, ^18^, ^19^, ^20^ ^]^ Larger and more complex ribozymes, such as novel group II introns (G2Is), have not yet been generated by these methods, to the best of our knowledge. This may be due to a limitation of the developed inverse folding algorithms to work with long sequences or due to a lack of understanding of the requisite composition of complex ribozymes that is needed for their de novo generation.

**Figure 1:**
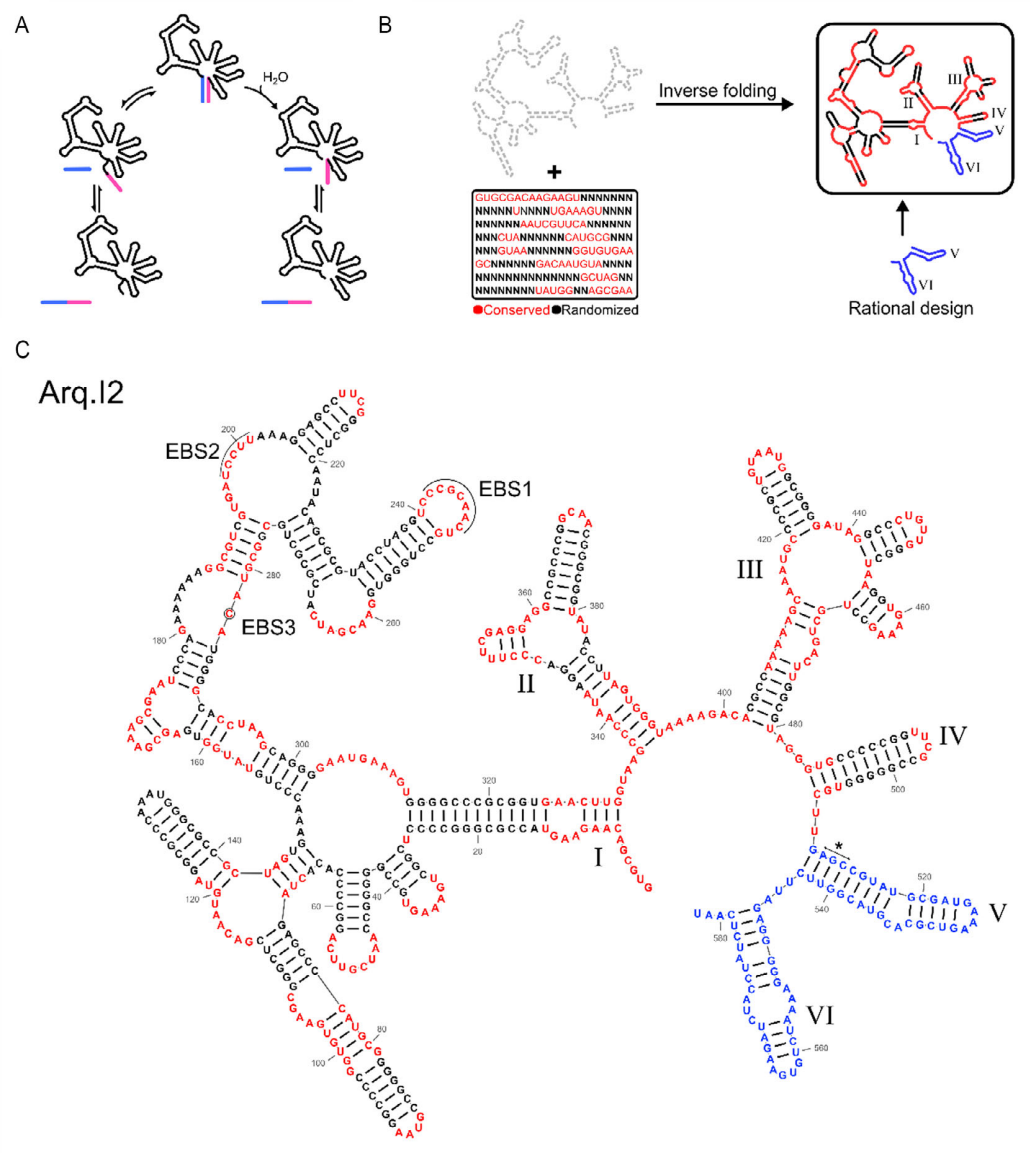
Design strategy of synthetic G2Is using inverse RNA folding. A) G2Is are self‐splicing ribozymes that catalyze their own excision from RNA transcripts through two alternative reaction pathways. In the branching pathway (left), the first nucleophilic attack is carried out by the bulged branch point adenosine, resulting in a lariat 3’ exon intermediate that makes the reaction fully reversible (left). In some cases, the first nucleophilic attack is carried out by a water molecule, resulting in a linear 3’ exon intermediate, making this step nonreversible (hydrolytic pathway, right).^[^ ^35^ ^]^ B) Inverse folding design strategy using aRNAque.^[^ ^13^ ^]^ The target secondary structures are based on the consensus structure of organellar class IIB1 intron domains I–IV. The sequence was constrained in areas of vital function and high natural conservation. Domains V and VI were rationally designed based on the most common motifs found in G2Is. C) Secondary structure diagram of Arq.I2. Sequences which were constrained during inverse folding are highlighted in red, sequence regions generated by aRNAque are shown in black, and rationally designed domains V and VI are highlighted in blue. The catalytic triad is indicated by an asterisk. EBSs 1–3 bind to respective IBS on the exons.

G2Is are self‐splicing ribozymes that catalyze their own excision from precursor transcripts and often act as mobile genetic elements, spreading to new genomic sites (**Figure** **1** A) by reverse splicing into DNA.^[^ ^21^ ^]^ Owing to their unique catalytic properties, G2Is show promise as biotechnological tools, such as for genome engineering. Mobile G2Is have been developed into “targetrons”, which can be reprogramed to insert into virtually any DNA sequence in prokaryotic cells with high specificity, with the assistance of their intron‐encoded protein (IEP).^[^ ^22^ ^]^ Over the past two decades G2I‐based targetrons have been successfully adopted for gene editing applications in bacteria.^[^ ^23^, ^24^ ^]^ More recent work has shown that protein‐free G2Is are capable of hydrolyzing dsDNA as well as inducing double‐stranded breaks in genomic DNA in vivo, which may lead to their development as compact, protein‐free genome editors for both prokaryotic and eukaryotic organisms.^[^ ^25^, ^26^ ^]^ In this work, anticipating a coming need to engineer custom G2Is, we have generated three active self‐splicing ribozymes using an inverse folding approach, which share little sequence identity with known G2Is.

We chose to do a more in‐depth biochemical characterization of one of the introns, Arq.I2, which showed unique properties not previously observed in naturally occurring G2Is, such as an unusually low predicted free energy of folding and little need for monovalent cations. In regard to its apparent rate of forward splicing, it finds its place among the fastest naturally occurring G2Is.^[^ ^27^, ^28^ ^]^ Remarkably, Arq.I2 even showed protein‐independent forward‐splicing activity in *Escherichia coli* cells. Our results serve as a proof of concept for generating large catalytic RNAs, which could be adapted to the generation of custom G2Is that meet specific needs. These could include G2Is carrying functional sequence elements optimized for purposes such as regulated gene expression, mRNA circularization, genome editing, RNA‐based therapeutics, and activity under extreme environmental conditions, opening new avenues for RNA‐based genome engineering and synthetic biology applications.

## 2 Results and Discussion

### 2.1 aRNAque Reliably Generates Novel Self‐Splicing Group G2Is

Designing a ribozyme de novo requires a clear understanding of the sequence and structural features that define this class of ribozymes. Although numerous G2Is have been identified based on conserved motifs, the features that enable efficient self‐splicing under physiological conditions remain poorly defined. To inform our design constraints, we chose to rely on sequence and structural conservation among closely related G2Is. The mitochondrial lineage of IIB1 G2Is emerged as an ideal candidate for this purpose. Notably, this lineage includes some of the most highly active and well‐studied G2Is, such as P.li.LSU.I2 from the brown alga *Pylaiella littoralis*.^[^ ^27^ ^]^ An additional advantage of this lineage is the availability of experimentally derived 3D structures, which facilitate the correlation of conserved sequence features with tertiary structures.^[^ ^29^, ^30^, ^31^, ^32^ ^]^

With the goal of identifying conserved sequence and structural features, we constructed a small library of mitochondrial G2Is through iterative BLAST searches of the RNA and protein components of IIB1 introns known to be self‐splicing under physiological conditions, and their secondary structures were predicted and analysed. The resulting collection of sequences (Supporting Information Dataset 1) and secondary structures provided insights into the permissible sequences and lengths of all stems, loops and junctions, as well as the relative presence or absence of certain subdomains or tertiary contacts. The analysis was further supplemented by consensus sequences generated using RNA‐specific multiple sequence alignment tools (Figure S1, Supporting Information Dataset 2).^[^ ^32^, ^33^ ^]^

Among the three secondary structure templates used as inverse folding targets, we kept the stems, loops, and junctions the same length as those found in P.li.LSU.I2, or the consensus if it was well conserved. We constrained sequence motifs with known functions in tertiary structure or catalysis (e.g., the internal loop of domain IC1, Figure S2, Supporting Information) to match the consensus sequence. We set the sequences of junctions with an unclear function and little sequence conservation to the length and sequence of the version found in P.li.LSU.I2. Similarly, in a bid to avoid the difficulties of RNA pseudoknot prediction, we fixed the sequence motifs of the α–α’ pseudoknot to match the ones found in P.li.LSU.I2. Lastly, we fixed the exon binding sites (EBSs) to match the ones previously used by our group.^[^ ^26^ ^]^ All other sequences in domains 1–4 that were believed to function only in establishing the correct secondary structure were randomized (Figure 1B). These sequence constraints and target structures were then used as an input for the RNA inverse folding software aRNAque.^[^ ^13^ ^]^ We chose this evolutionary algorithm because its mutation scheme, inspired by Lévy flights,^[^ ^34^ ^]^ is optimized to explore complex energy landscapes while minimizing trapping in local minima or plateaus.

In order to reduce execution time, we subjected only domains I–IV (D1234) to inverse folding, whereas domains V and VI (D56), which are short, highly conserved, independently folding domains^[^ ^35^, ^36^ ^]^ were rationally designed based on the most common sequence features observed in the intron library. We ran aRNAque a total of three times, each time varying the RNA structure, sequence constraints, and command‐line arguments slightly, in the hopes of exploring a greater diversity of sequence outputs. In total, the sequence constraints amounted to 239/582 (41%) randomized positions in iteration 1 and 2, and 229/569 (40.2%) randomized positions in iteration 3. We selected the best‐scoring D1234 sequences in each iteration evaluated according to their normalized energy distance (NED),^[^ ^37^ ^]^ and appended the rationally designed D56 domain to their 3’ ends (Figure 1B), resulting in the three synthetic G2Is Arq.I1, Arq.I2, and Arq.I3 (Figure 1C, Figure S3 and S4, Supporting Information).

All three sequences were considerably dissimilar to P.li.LSU.I2, which was used as the primary template for their design. Their sequence identity to P.li.LSU.I2 (Arq.I1 = 60.3%, Arq.I2 = 57.9%, Arq.I3 = 58.4%) was comparable to that of the related intron P.li.LSU.I1 (56.7%), or *Scenedesmus obliquus* LSU.I1 (65%)^[^ ^38^ ^]^ (Table S2, Supporting Information Dataset 3) classifying them as distinct de novo representatives of the mitochondrial lineage of class IIB1 G2Is. Unexpectedly, a comparison of the minimum free energies of folding of Arq.I1, Arq.I2, and Arq.I3 with other G2Is predicted extraordinarily stable folds of the three computationally designed ribozymes compared to their natural counterparts (Table S3, Figure S5, Supporting Information).

**Figure 2:**
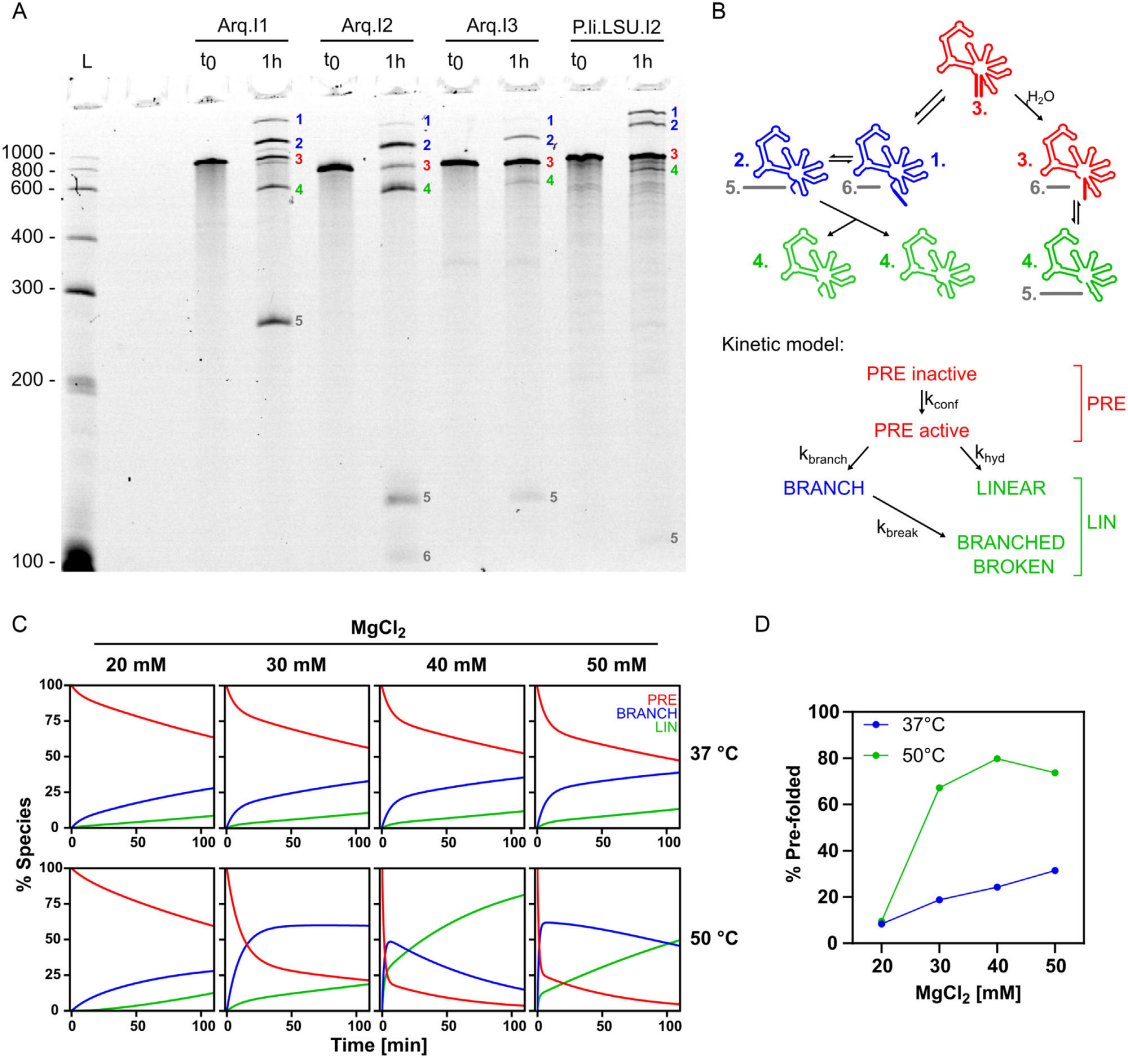
In vitro characterization of the self‐splicing activity of the computationally designed G2Is. A) 5% urea‐PAGE showing the splicing products of computationally designed introns Arq.I1, Arq.I2, and Arq.I3 alongside splicing products of naturally occurring G2I P.li.LSU.I2 for comparison.^[^ ^27^ ^]^ Introns were incubated for 1 hr at 47 °C in splicing buffer containing 40 mM Tris‐HCl pH 7.5, 0.05% Tween 20, 50 mM (10 mM for P.li.LSU.I2) MgCl_2_ and 250 mM (100 mM for Arq.I3) NH_4_Cl. Different species are annotated by numbers on the gel, of which the corresponding species are shown in (B). B) Top: Schematic of the splicing reaction. The species are numbered the same way as in the gel in panel (A). Bottom: Schematic of the kinetic model. The species used for the kinetic models in (C) are annotated and highlighted by color: BRANCH is shown in blue, PRE in red, and LIN in green. C) Kinetic models of forward splicing reactions. Reactions were incubated at 37 °C or 50 °C and in varying concentrations of MgCl_2_ (20, 30, 40, and 50 mM). D) Percentage of prefolded population at varying reaction conditions.

Since the in silico results did not provide any information about the catalytic properties of the new G2Is, we synthesized all three ribozyme candidates and tested their in vitro self‐splicing activity. Remarkably, all three introns were capable of autocatalytic self‐splicing under conditions routinely used for such assays, namely a buffer containing Tris‐HCl pH 7.5, Tween 20 and varying concentrations of MgCl_2_ and NH_4_Cl (**Figure** **2**A). We observed the formation of the canonical intron lariat and spliced exons for all three designed introns. Splicing of Arq.I1 and Arq.I2 also resulted in the emergence of the lariat‐3’exon intermediate and a band which could correspond to the linear intron (the product of hydrolytic splicing) or the broken lariat (Figure 2B). Interestingly, under these conditions, both Arq.I1 and Arq.I2 spliced to a greater degree than the P.li.LSU.I2 intron, which was mutated to share the same EBSs as the designed introns. As this was an endpoint experiment, it does not provide information on the reaction rates. However, as the modified P.li.LSU.I2 is expected to splice relatively quickly, it is likely that the reaction reached equilibrium within 1 h.^[^ ^27^ ^]^ The most probable explanation for the reduced splicing efficiency observed for the P.li.LSU.I2 variant is that altering its EBSs has shifted the chemical equilibrium toward reverse splicing. Nevertheless, the fact that it was seemingly outperformed by Arq.I1 and Arq.I2 is unexpected, considering that the wild‐type intron is reported to be the fastest splicing G2I discovered thus far.^[^ ^27^, ^39^, ^40^ ^]^ In spite of the small number of ribozymes designed and tested, the fact that all three of the introns readily self‐spliced indicates that the method used to design them is robust and can reliably produce functional group IIB1 introns.

The success of this design approach is likely due to the cautious choice of sequence constraints, which aimed to ensure that the designed sequences lacked no functional motifs, and that these motifs deviated as little as possible from those seen in well‐studied introns or the consensus sequence. While aRNAque performed well at these design tasks, we expect that other inverse folding algorithms that can handle long sequences would be comparably successful in generating G2Is with the constraints we used in this study. One limitation of the latest version of aRNAque in terms of ribozyme design is a lack of support for the International Union of Pure and Applied Chemistry degenerate nucleotide code other than A, G, U, C, and N. We expect that artificial G2Is could be designed to target virtually any exon sequences, as has been the case with targetrons and HYERs.^[^ ^25^, ^41^ ^]^ While generally retargetable, there is variance in the effectiveness of different EBSs, that does not follow a clear pattern such as GC content.^[^ ^25^, ^41^ ^]^ Most likely these differences are caused by differential metal ion coordination in the EBS:intron binding sites (IBS) major grooves,^[^ ^42^, ^43^ ^]^ or possibly as a result of misfolding. In this regard, G2Is designed de novo might outperform targetrons derived from naturally occurring G2Is because the design process allows them to be fine‐tuned so that they do not misfold with any arbitrary EBS sequences.

### 2.2 Biochemical Characterization of an Artificial Group II Intron

Because Arq.I2 demonstrated the highest apparent reactivity in initial experiments, as well as having been predicted to have an unusually stable secondary structure (Figure S5, Supporting Information), it was chosen for a more in‐depth characterization. To this end, we performed reaction time‐course analyzes at increasing MgCl_2_ concentrations at either 37 or 50 °C (Figure S6, Supporting Information).

The complexity of the G2I forward‐splicing reaction means that modeling it is not straightforward. Canonically it involves the catalysis of two consecutive reversible transesterifications, separated by a large conformational change.^[^ ^31^ ^]^ Fully modeling all known G2I reactions is outside of the scope of this work, and we chose to reduce complexity as much as possible (for a more in‐depth description of the kinetic model, please refer to the Supporting Information). As such, all branched products were treated as a single species, and all reactions were modeled using pseudoirreversible apparent rate constants.

In our gel‐based assays, we observed a slow accumulation of linear products alongside an equal and simultaneous decrease in branched species at later time points. We attributed this to the nonenzymatic cleavage of the RNA backbone in branched species resulting in molecules that comigrate with the linear intron^[^ ^44^ ^]^ and were modeled accordingly. Although the potential contribution of the specific hydrolysis of the 2'‐5' linkage^[^ ^45^ ^]^ to this band cannot be discounted, to the best of our knowledge it has not been reported to occur in full‐length introns, and our data was fit well by a single reaction converting the branched species to linear species. Since the biphasic depletion of the precursor observed in all reactions could not be explained solely by the combined action of the branching and hydrolysis reactions, we concluded that this behavior is most likely caused by the conformational change of domain 6 (D6) that is responsible for the exchange of splice sites in the active site.^[^ ^30^ ^]^ It has been observed early on that the conformational state of D56 resulted in two‐phase kinetics.^[^ ^45^ ^]^ We model this as a percentage of the population being primed (or correctly “prefolded”) for branching because they fold directly into the conformation in which the ι–ι′ interaction is engaged. The remaining introns fold into the conformation in which the π–π′ and η–η′ interactions are engaged, and these introns must first undergo a slow, rate‐limiting conformational change to the branching conformation.

Our kinetic model, which was fitted to the time‐course data, reproduces the observed reaction profiles well (Figure S6, Supporting Information) and supports the interpretation that the biphasic kinetics are caused by structural toggling of D6. The initial burst in fast splicing can be attributed to a population of introns that is already in the branching conformation. Meanwhile, at later time points, a second, much slower Mg^2+^‐independent conformational change becomes rate limiting for splicing.

Intriguingly, although previous studies have shown that both the rate of folding and catalysis of the Sc.ai5γ intron are positively affected by Mg^2+^ concentration,^[^ ^46^ ^]^ this does not seem to be the case for Arq.I2 at 37 °C. Instead, a model constraining all reaction rates to be identical at all MgCl_2_ concentrations fits the data well (Figure 2C, Table S4, Supporting Information). At this temperature, the only effect of increasing MgCl_2_ concentrations seems to be in increasing the fraction of introns that begin in the branching conformation. This is reminiscent of the finding that the ratio of fast to slow reacting introns was controlled by the strength of the π–π′ and η–η′ interactions and Mg^2+^ concentration.^[^ ^39^ ^]^ Certain variants of P.li.LSU.I2 with mutated D2:D6 tertiary contacts (π–π′ and η–η′) reacted only with a slow reaction phase at low Mg^2+^ concentrations.^[^ ^39^ ^]^ Interestingly, biphasic kinetics in these introns were restored at elevated Mg^2+^ concentrations which reactivated the fast‐splicing pathway.^[^ ^39^ ^]^ Taken together with our results, this seems to indicate that the [Mg^2+^]_½_ of the ι–ι′ interaction, which positions D6 for branching, is considerably higher than the π–π′ and η–η′ interactions, which are engaged during exon ligation. According to this framework, the strength of the ι–ι′ interaction increases with Mg^2+^ concentration, whereas the strength of the π–π′ and η–η′ interactions is less affected. This results in a larger percentage of introns starting out with the ι–ι′ interaction engaged at *t* = 0 and being primed for branching at elevated Mg^2+^ concentrations, as seen in the case of Arq.I2 at 37 °C (Figure 2D and S6, Supporting Information).

However, this does not seem to be the case at 50 °C, where, in addition to this effect, Mg^2+^ also influences the reaction rates, with an optimum for the apparent rate of branching at around 50 mM MgCl_2_ (*k*_branch_ = 0.8 min^−1^, Table S4, Supporting Information), whereas the apparent rate of hydrolytic splicing seems to be highest around 40 mM Mg^2+^ (*k*_hyd_ = 0.23 min^−1^, Table S4, Supporting Information). At 50 °C the reaction rates decrease markedly below 40 mM MgCl_2_, indicating that higher concentrations of divalent cations are required to stabilize the native tertiary fold at elevated temperatures, as also observed for P.li.LSU.I2.^[^ ^27^ ^]^ It must be noted that our experimental setup cannot resolve whether folding or catalysis is rate limiting, and therefore it is unclear whether the measured rates of branching reflect the rates of chemistry or tertiary folding.

Although our model provides valuable insights into the inner workings of the Arq.I2 self‐splicing reaction, it does not permit direct comparisons with other G2Is described in the literature. To address this, we determined the apparent precursor depletion rates by fitting our data to the standard irreversible two‐phase model for direct comparison with rates of naturally occurring G2Is determined elsewhere.^[^ ^27^, ^28^ ^]^ According to this model, Arq.I2 has a *k*_fast_ = 1.83 min^−1^, placing it among some of the fastest reacting naturally occurring G2I discovered to date. It is almost 50‐fold faster than the model intron Sc.ai5γ (*k*_fast_ = 0.038 min^−1^),^[^ ^47^ ^]^ roughly on par with recently discovered highly reactive G2Is (C.i.LSU.I3: *k* = 2.0 min^−1^ and C.i.SSU.I1: *k*_fast_ = 2.94 min^−1^),^[^ ^28^ ^]^ but still slightly slower than the fastest discovered G2I, P.li.LSU.I2 (*k*_fast_ = 5 min^−1^).^[^ ^27^ ^]^ It is remarkable that such a fast ribozyme could be identified from as few as three designs. Typically, ribozyme design campaigns using similar approaches require screening of a large number of candidates and result in ribozymes which generally fall short of the catalytic rates of wild‐type ribozymes.^[^ ^18^, ^19^, ^20^ ^]^

The aRNAque algorithm^[^ ^13^ ^]^ robustly generated highly active G2Is with unusually stable secondary structures. The three designed introns are moderately GC‐rich (Arq.I1: 56% GC; Arq.I2: 59.5% GC; Arq.I3: 58% GC), which is a common feature among natural introns. Certain IIB1 introns, such as Rh.op.I1, and class G G2Is have GC contents of up to 65% or more.^[^ ^48^ ^]^ Whether this elevated GC content contributes to ribozyme activity remains unclear. Notably, Arq.I2 displays efficient self‐splicing even at low monovalent salt concentrations and even retains measurable catalytic activity in the absence of added monovalent salts (Figure S7, Supporting Information), a property that may also apply to naturally occurring GC‐rich introns.

Importantly, all aRNAque‐designed introns appear to reliably fold into their predicted (and intended) secondary structures. In contrast, the majority of naturally occurring G2Is are predicted to misfold by RNA folding algorithms, raising the question of why natural selection has not consistently selected for such robust folding. One plausible explanation is that highly active introns may be counter‐selected due to their deleterious proliferation in host genomes, leading to extinction dynamics.^[^ ^49^ ^]^ As a result, genomes harboring G2Is often contain only a few copies, and less active introns may be overrepresented.

A second, nonexclusive explanation is based on neutral evolution,^[^ ^50^, ^51^ ^]^ whereby G2Is coevolve with their IEPs that act as chaperones facilitating intron folding.^[^ ^52^, ^53^ ^]^ Therefore, mutations in the ribozyme that impair folding can thus be masked by the IEP cofactor and allow structurally suboptimal variants to persist, a phenomenon known as presuppression.^[^ ^50^, ^51^ ^]^ We have previously argued that this may explain why most G2Is are inactive in the absence of their IEP.^[^ ^26^ ^]^ While such neutral models are particularly compelling,^[^ ^54^ ^]^ both evolutionary dynamics, purifying selection, and presuppression likely shaped the sequence space of modern G2Is.

### 2.3 Arq.I2 Self‐Splices in TXTL Reactions and *E. coli* Cells

**Figure 3:**
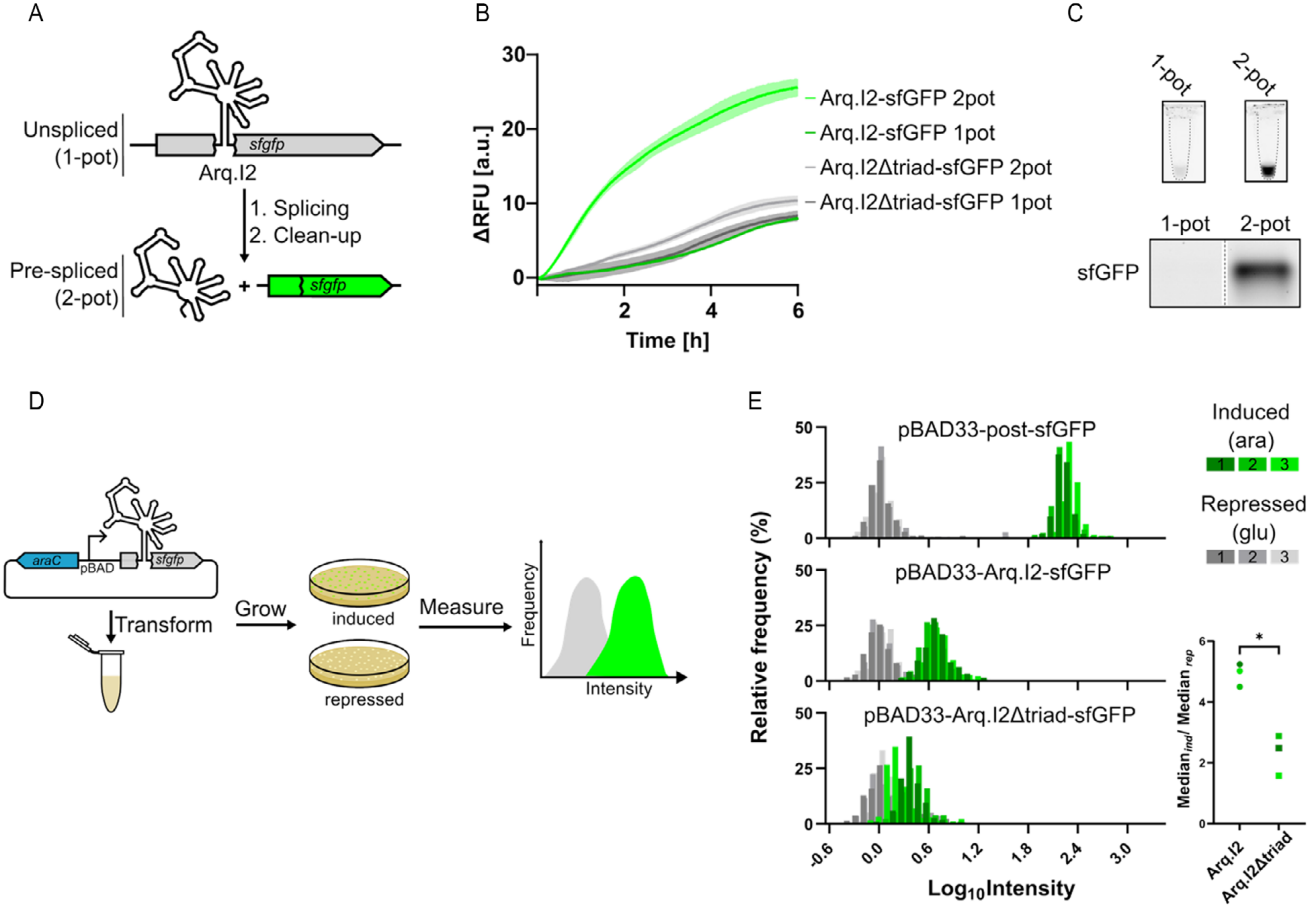
Arq.I2 splicing yields functional mRNA in TXTL reactions and *E. coli* cells. A) A pre‐mRNA construct containing the coding sequence for sfGFP interrupted by Arq.I2 was added to the PURExpress TXTL system. The sample was either incubated for 1 hr at 50 °C in splicing buffer containing 350 nM RNA template, 100 mM MgCl_2_, 250 mM NH_4_Cl, 0.05% Tween 20, and 40 mM Tris‐HCl pH 7.5 (prespliced/2‐pot) or added directly to the PURE reaction (unspliced/1‐pot). B) Fluorescence time traces (520 nm) of PURExpress reactions incubated at 37 °C containing either untreated Arq.I2‐sfGFP or Arq.I2Δtriad‐sfGFP RNA (one‐pot) or prespliced RNA (two‐pot). Experiments were carried out in independent triplicates). C) Top: Endpoint fluorescence of PURExpress reaction tubes scanned at 488 nm excitation/520 nm emission. Bottom: 10% SDS‐PAGE of the PURE reactions scanned at 488/520. D) Plasmids containing the coding sequence for sfGFP interrupted by either Arq.I2 or Arq.I2Δtriad were transformed into *E. coli* cells. The pBAD‐33‐postsfGFP construct was used in cells as a positive control to simulate the maximum possible signal from 100% splicing activity. Cells were grown overnight on agar plates containing either 0.2% arabinose (induced) or 0.2% glucose (repressed) and scanned at 488/520 to quantify sfGFP production. E) Histogram showing the distribution of signal intensities among colonies grown on agar plates containing arabinose (ara) or glucose (glu). The panel on the right shows the colony fluorescence median ratios from arabinose‐ (Median_ind_) and glucose‐ (Median_rep_) supplemented plates for cells carrying either pBAD‐33‐Arq.I2‐sfGFP or pBAD‐33‐Arq.I2Δtriad‐sfGFP (*N* = 3 independent replicates, *α* = 0.05 using a one‐sided Mann–Whitney U‐test).

Considering that Arq.I2 retains activity at low concentrations of monovalent cations, as well as at moderate temperatures, we next sought to investigate whether the intron might be active under physiological conditions. To do this, we tested its ability to produce functional mRNA in PURExpress, a recombinant in vitro transcription–translation (TXTL) system based on *E. coli*.^[^ ^55^ ^]^ We designed a precursor mRNA transcript in which the coding sequence for the reporter protein sfGFP is interrupted by the Arq.I2 intron, making protein expression dependent on successful self‐splicing and exon ligation (**Figure** **3**A). The Arq.I2 sequence, including the necessary intron binding sites, were inserted after the second codon (Ser2) of the sfGFP open reading frame. The Arq.I2 sequence contains multiple stop codons in all three reading frames, preventing translation of fluorescent sfGFP from unspliced transcripts.

As an initial test of Arq.I2's ability to produce functional mRNA, the unspliced transcript was preincubated for 1 h in splicing buffer containing 100 mM MgCl_2_, followed by column purification and resuspension in H_2_O prior to its addition to the PURExpress reaction (“2‐pot”, Figure 3A). Under these conditions, PAGE analysis confirmed that Arq.I2 could successfully catalyze forward splicing and exon ligation from the sfGFP mRNA (Figure S8, Supporting Information). In agreement with this, the two‐pot sample showed an increase in fluorescence in the TXTL reaction after a short lag phase of ≈15 min, indicating immediate transcription and sfGFP expression followed by maturation of the GFP fluorophore (Figure 3B). We could confirm that the observed increase in fluorescence resulted directly from exon ligation of the sfGFP mRNA since only background levels of fluorescence were observed when RNA constructs with a mutated catalytic triad in domain V (Arq.I2Δtriad) were used as templates in TXTL reactions. To further exclude that the increase in emission was a result of background autofluorescence or light‐scattering, the presence of functional sfGFP was further confirmed by direct in‐tube fluorescence imaging as well as in‐gel fluorescence imaging after semi‐denaturing sodium dodecyl sulfate–polyacrylamide gel electrophoresis (SDS‐PAGE) (Figure 3C, Figure S9 and S10, Supporting Information).

Encouraged by these results, we wondered if Arq.I2 was also active directly in the PURE system, which contains low, near‐physiological levels of free Mg^2+^ ions.^[^ ^56^ ^]^ In these “1‐pot” experiments, the unspliced transcript was added directly to the PURE reaction prior to incubation and real‐time sfGFP fluorescence recording at 37 °C (Figure 3A). However, no difference in fluorescence was observed between samples containing sfGFP mRNA interrupted by either active Arq.I2 or inactive Arq.I2Δtriad (Figure 3B). Furthermore, sfGFP expression from one‐pot reactions could not be detected via in‐tube or in‐gel imaging. In contrast, targeted triple quadrupole LC‐MS/MS analysis detected multiple sfGFP‐specific peptides in the Arq.I2 sample (Figure S11; Tables S5 and S6, Supporting Information). This finding suggests that low but detectable levels of self‐splicing and exon ligation occur even under the stringent, the low‐Mg^2^^+^ conditions of the TXTL system, enabling a small degree of downstream reporter expression.

Finally, we set out to probe whether the artificial G2I Arq.I2 could also be active in living cells. In intracellular environments, weakly chelated Mg^2+^ ions are present at upper millimolar concentrations, which can promote ribozyme catalysis even when free Mg^2+^ is limited.^[^ ^57^ ^]^ To assess whether such conditions are compatible with Arq.I2 self‐splicing, we constructed an arabinose‐inducible plasmid encoding sfGFP interrupted by the Arq.I2 sequence, rendering reporter expression dependent on successful self‐splicing (pBAD33‐Arq.I2‐sfGFP). As a negative control, we created the same construct using catalytically inactive ArqI2Δtriad. The plasmids were transformed into chemically competent *E. coli* cells and plated on agar plates supplemented with either 0.2% arabinose (inducing) or 0.2% glucose (repressing transcription).^[^ ^58^ ^]^ After overnight incubation at 37 °C, sfGFP fluorescence was quantified in individual colonies at 520 nm following excitation at 480 nm (Figure 3D; see Supporting Information for details).

For both constructs, colony fluorescence was stronger on induced compared to repressed plates. However, cells carrying the active Arq.I2 construct consistently showed modest but statistically significant increases in green fluorescence upon induction relative to the inactive control (Figure 3E), indicating low levels of successful splicing and reporter expression. At the same time, intracellular sfGFP expression from the induced Arq.I2‐sfGFP construct remained below the levels observed with a positive control encoding the fully spliced reporter transcript (pBAD‐33‐post‐sfGFP, Figure 3E), similar as observed in vitro (Figure S12, Supporting Information). The small fluorescence detected for the induced inactive Arq.I2Δtriad construct is likely attributable to stress‐induced autofluorescence, a phenomenon commonly observed in *E. coli* under metabolic stress.^[^ ^59^ ^]^ Despite these limitations, detectable fluorescence confirms that Arq.I2 retains catalytic activity in living cells.

Taken together, our results demonstrate that Arq.I2 can fold and self‐splice in vitro and exhibits low but measurable activity under intracellular conditions. This limited in vivo activity is reminiscent of other protein‐free G2Is^[^ ^53^ ^]^ and suggests that, with further optimization, for example, via directed evolution, artificial G2Is may also achieve robust function in intracellular environments.

## 3 Conclusions

In this study we used RNA inverse folding to design self‐splicing G2Is that are each substantially different from any known G2I found in nature. Extensive secondary structure optimization by aRNAque's evolutionary algorithm produced ribozymes with an unusually stable secondary structure (Figure S5, Supporting Information), and consequently, a minimal requirement for monovalent salts (Figure S7, Supporting Information). The ribozyme chosen for detailed characterization, Arq.I2 (Figure 1C), achieved a rate of self‐splicing comparable to the fastest naturally occurring G2Is discovered. Under in vitro conditions, Arq.I2 was found to self‐splice from transcripts encoding a reporter protein, which could then be used for reporter gene expression in a cell‐free TXTL system (Figure 3). While its intracellular activity is currently limited, further optimizations, for example, by in vivo evolution, might unlock higher levels of in vivo activity. The size and complexity of the G2Is designed in this study far exceed those of previously designed ribozymes.^[^ ^17^, ^18^, ^19^, ^20^ ^]^ These results serve as a proof of concept for the de novo generation of other large and complex ncRNAs. We expect that comparable success can be achieved with the design of other classes of G2Is, group I introns, and possibly even ribosomal RNAs,^[^ ^60^, ^61^, ^62^ ^]^ or other noncatalytic ncRNAs, provided that sufficient care is taken with regards to the sequence constraints. Considering the renewed interest in G2Is as protein‐free biotechnological tools,^[^ ^25^, ^26^, ^63^, ^64^ ^]^ these results open the door to the design of bespoke G2I‐derived ribozymes. Future ribozymes generated by inverse folding approaches may include catalysts for protein‐free gene editing, RNA processing, circRNA production, or therapeutics with reduced immunogenicity. Advances in RNA 3D structure prediction are expected to greatly improve the efficacy of the design process,^[^ ^65^ ^]^ while novel machine learning approaches show great promise for RNA design.^[^ ^66^, ^67^, ^68^, ^69^ ^]^

## 4 Experimental Section

4.1

4.1.1

##### DNA Templates

All DNA oligos and gene blocks were purchased from Integrated DNA Technologies (IDT). Templates for in vitro transcription were generated by polymerase chain reaction (PCR) performed in 100‐μl reactions using Q5 Hot Start High‐Fidelity 2x Master Mix (New England Biolabs ‐ NEB), 0.5 μM of each primer and 1 ng of template. PCR products were purified using the Monarch PCR & DNA Cleanup Kit (NEB) and analyzed on a TapeStation system (Agilent) or an agarose Tris‐acetate‐EDTA (TAE) gel to confirm their size. Template concentration and purity were determined by measuring the absorbance at 260 nm using a Nanodrop spectrophotometer (Thermo Fisher Scientific).

##### Computational Design of G2Is

A small library of G2Is related to P.li.LSU.I2 and belonging to the organellar class IIB1 introns was assembled through iterative BLAST searches^[^ ^70^ ^]^ starting with the ribozyme and IEP components of some highly active introns.^[^ ^27^, ^28^ ^]^ When present, the open reading frames were truncated, and the RNAs folded into their predicted secondary structures using the *mfold* web server^[^ ^71^ ^]^ and the appropriate folding constraints of the intron class.^[^ ^72^ ^]^ Consensus sequences were also generated using both the rMSA^[^ ^33^ ^]^ and RNA‐MSM^[^ ^73^ ^]^ web servers using domains 1–3 of P.li.LSU.I2 as the query. The combined secondary structure library, the available 3D models,^[^ ^29^, ^30^, ^31^ ^]^ and the consensus sequences informed the design process, which utilized the 2022 version of the RNA inverse folding algorithm aRNAque,^[^ ^13^ ^]^ which was installed from its GitHub repository (https://github.com/lemerleau/aRNAque). The algorithm was used to generate a number of candidate sequences proposed to fold into the target secondary structure, of which the top‐scoring sequence was chosen for experimental validation. The introns Arq.I1, Arq.I2, and Arq.I3 were the result of three different aRNAque runs with different secondary structure and sequence constraints. All three runs generated only intron domains 1–4 (D1234). For the full aRNAque command‐line arguments used, please refer to the supplementary methods. The introns were completed with the addition of a rationally designed pair of domains 5 and 6 (D56). The high degree of conservation in D56 obviated the need for inverse folding, allowing for the design of these domains according to their consensus (Table S1, Supporting Information).

##### RNA Preparation

In vitro transcription of intron RNA and IVS‐sfGFP constructs was performed using the TranscriptAid T7 high yield transcription kit (Thermo Fisher Scientific) according to the manufacturer's instructions, with the addition of 2.5 μM Cy5‐tagged UTP (pyrimidine nucleoside triphosphate) only in the transcription reaction of introns with noncoding exons (0.025% of total UTP in reaction). After incubation for 4 h at 37 °C, the DNA template was digested in IVT buffer by addition of 1 μL of Turbo DNAse (Thermo Fisher Scientific) and incubated for 30 min. Samples were then purified using the Monarch RNA Cleanup Kit (NEB), and the RNA concentration was determined by measuring absorbance at 260 nm on a Nanodrop spectrophotometer (Thermo Fisher Scientific). The correct product size was confirmed by electrophoresis on a 4.5% denaturing urea‐PAGE (19:1 acrylamide:bisacrylamide, 8 M urea).

##### Forward Splicing Reactions

All splicing reactions were performed in 40 mM Tris‐HCl (pH 7.5), 0.05% v/v Tween 20, 0.25 M NH_4_Cl, 1.5 μM intron RNA, and varying concentrations of MgCl_2_. Prior to initiating the reactions, the intron RNA was refolded in a buffer containing Tris‐HCl and Tween 20 by heating to 90 °C for 2 min, followed by slow cooling to 25 °C (0.5 °C s^−1^) and then transferred to ice. Samples for the *t* = 0 time points were collected and mixed with NH_4_Cl and MgCl_2_ stocks on ice. The remaining reaction mixture, NH_4_Cl and MgCl_2_ stocks were then brought to the target temperature (37 °C or 50 °C) on a thermocycler and mixed together to initiate the reaction. For PAGE analysis (see below), 2 μL of forward splicing reactions were taken at increasing time points for gel electrophoresis and quenched in 68 μL of denaturing gel loading buffer (98% formamide, 10 mM ethylenediaminetetraacetic acid (EDTA) pH 8, and cresol red tracking dye). Care was taken to ensure that the amount of MgCl_2_ was always exceeded by EDTA, and the samples were stored at −20 °C. All experiments were performed in triplicate (*n* = 3).

##### Denaturing PAGE Analysis

Gels (10 × 10 × 0.1 cm) were prepared by mixing the desired ratio of stock solutions containing 8 M urea, 1× TBE (Tris‐borate‐EDTA), and either 20% or 0% acrylamide. Gel casting stock solutions contained a 19:1 ratio of acrylamide to bisacrylamide and were filtered and degassed. Electrophoresis was performed using 1× TBE as the running buffer. To permit the denaturation of Arq.I2, both the gels and TBE‐Buffer were preheated to 65 °C before sample loading. Electrophoresis was carried out on 1 mm‐thick polyacrylamide gels at a constant power of 10 W until the cresol red front reached the bottom of the gel. Gels were subsequently imaged using a Sapphire Biomolecular Imager (Azure Biosystems).

##### Preparation of Arq.I2‐SfGFP Constructs

A pBAD33 plasmid containing the sfGFP coding sequence was used as the starting point for the generation of the construct containing the Arq.I2 presplicing sequence in the sfGFP open reading frame after the second codon (pBAD33‐Arq.I2‐sfGFP). The construct was generated by HiFi DNA assembly using a 758‐bp g‐block from IDT as an insert according to the supplier`s instructions. A postsplicing control construct (pBAD33‐postsfGFP), which contains the expected sequence after Arq.I2 splicing, was generated by PCR using pBAD33‐Arq.I2‐sfGFP as a template. Forward and reverse primers were designed to exclude the intervening Arq.I2 sequence while retaining the intron binding sites. The catalytically inactive variant Arq.I2Δtriad (harboring the mutations A511G, G512A, and C513U) was introduced into the pBAD33‐Arq.I2‐sfGFP construct by site‐directed mutagenesis PCR using the primers listed in Table S1, yielding pBAD33‐Arq.I2Δtriad‐sfGFP. Blunt‐ended PCR products were generated using the Q5 High‐Fidelity 2x Master Mix (NEB), followed by 5′‐phosphorylation, recircularization, and DpnI digestion using the KLD enzyme mix (NEB). The sequences of all constructs were confirmed by Sanger sequencing.

For the TXTL experiments, linear DNA templates containing a 5’‐terminal T7 promoter and either the Arq.I2‐sfGFP or the Arq.I2Δtriad‐sfGFP coding sequence were generated by PCR. A forward primer containing the T7 promoter sequence in the overhang (Table S1), binding upstream of the ribosome binding site, and a reverse primer binding downstream of the sfGFP sequence were used. The resulting linear constructs of 1557 bp, or 975 bp in the case of the positive control, were purified using the Monarch DNA & PCR Cleanup Kit (NEB), and their concentration was determined by measuring absorbance at 260 nm using a Nanodrop spectrophotometer (Thermo Fisher Scientific). For primer and plasmid sequences, please refer to Table S1 in the Supplementary Information.

##### in vitro Transcription Translation Reactions

Coupled in vitro TXTL reactions were carried out using the commercially available PURExpress system (NEB). Typically, 12.5 μL of TXTL reactions were prepared using 5 μL of PURExpress Solution A, 3.75 μL of PURExpress Solution B, 1 U of Murine RNase Inhibitor (NEB), and either 450 or 350 nM of the linear Arq.I2‐sfGFP RNA or 12.5 nM of the linear positive control DNA. In the case of the ‘linear prespliced’ samples, the Arq.I2‐sfGFP RNA construct was allowed to splice under standard forward splicing conditions (50 μL reactions supplemented with 100 mM MgCl_2_) at 50 °C for 1 h prior to TXTL. Prior to addition to the reaction, the processed RNA was purified using the Monarch RNA Cleanup Kit (NEB) and eluted in 10 μL to avoid buffer interference with the PURE system and to concentrate the splicing product. Samples were incubated at 37 °C for 6 h, while fluorescence was measured in a StepOne qPCR machine (Applied Biosystems) at 1 min intervals. After incubation, reaction tubes were additionally imaged on a Sapphire Biomolecular Imager (Azure Biosystems) at 488/520 nm to confirm the presence of sfGFP. For in‐gel imaging, TXTL samples were heat‐denatured at 60 °C for 10 min in Laemmli loading buffer to prevent decomposition of the GFP‐fluorophore before 10% SDS‐PAGE. The gel was imaged at 488/520 nm to visualize the sfGFP and 658/710 nm to visualize the Prestained BlueClassic Protein Ladder (Jena Bioscience). Experiments were performed in triplicate (*n* = 3).

##### Expression of Arq.I2‐sfGFP Constructs in E. coli

To probe Arq.I2 activity in *E. coli* cells, 1 ng of either pBAD33‐Arq.I2‐sfGFP, pBAD33‐Arq.I2Δtriad‐sfGFP, or pBAD33‐postsfGFP were transformed in chemically competent Top 10 *E. coli* cells and grown overnight on agar plates containing chloramphenicol (100 μg ml^−1^) and either 0.2% of arabinose, to activate transcription, or glucose, to repress transcription, respectively. Plates were imaged on a Sapphire Biomolecular Imager (Azure Biosystems) at 488/520 nm and analyzed using ImageJ to quantify sfGFP expression. For a detailed description of the image processing, please refer to the Supplementary Information. Experiments were performed in independent triplicates (*n* = 3).

## Conflict of Interest

The authors declare no conflict of interest.

## Author Contributions

**Deni Szokoli** conceived the computational design workflow for G2I *de novo* design, performed the bioinformatic analysis of the input sequences, designed the introns, supervised parts of the study, designed the experiments, analysed the data, performed the kinetic modelling and wrote the manuscript. **Noemi E. Nwosu** designed the experiments, performed the biochemical characterization, collected and analysed the data for the splicing experiments in the TX‐TL system and in *E. coli* cells, prepared the figures and wrote the manuscript. **Lukas M. Glatt** performed the biochemical characterization experiments. **Hannes Mutschler** supervised and coordinated the study, designed and conceived experiments and wrote the manuscript. **Deni Szokoli** and **Noemi E. Nwosu** contributed equally to this work and treated as co‐first authors.

## Supporting information

Supplementary Material

## Data Availability

The data that support the findings of this study are openly available in [Figshare] at [https://doi.org/10.6084/m9.figshare.29262671], reference number [29262671].
