# Supplementary material for: Computational De Novo Design of Group II Introns Yields Highly Active Ribozymes
Source: Chembiochem. 2025 Jun 30;26(14):e202500356. doi: 10.1002/cbic.202500356 (PMC12278348; doi:10.1002/cbic.202500356)
Supplement: Supplementary file 1 — Supplementary Material [file CBIC-26-e202500356-s001.pdf]

ChemBioChem

Supporting Information

Computational *De Novo* Design of Group II Introns Yields Highly Active Ribozymes

Deni Szokoli[+], Noemi Ezinne Nwosu[+], Lukas Maria Glatt, and Hannes Mutschler[*]

Content

Supplementary methods **3**

Inverse folding 3

Molar fractions of RNA species from PAGE experiments 4

Model fitting 4

Statistics 5

Alignments 5

Image processing using ImageJ 6

Trypsin digest of PURExpress reactions 6

Proteomic analysis 7

Supplementary Figures **8**

Supplementary Tables **17**

References **36**

Supplementary methods

Inverse folding

The intron Arq.I1 was generated using the command:

```
python aRNAque.py -g 100 --verbose --
target="......((((...(((((((((((((.((((.......))))((((((.........)))))).(((
((((((((((..(.((((((....)))))).)..))....)))))).........(((((((....)))))))..
)))))...((((((..((((.((....))...(((((........((((((...........((((((....)))
)))....((((((((((((((((.........)))))))))..........)))))))))))))...))))))))
)...))))))..........)))))))))))))..))))....(((((.(((((..(((....)))..(((((((
(....))))))))...))))).)))))........(((((((..((.....((((((((...))))))))...((
(((....))))).((((....))))))...)))))))....((((((((((....))))))))))..." --
C=GUGCGACAAGAAGUNNNNNNNNNNNNNUNNNNNUGAAAGUNNNNNNNNNNNNAAUCGUUCANNNNNNNNNNCUANNNN
NNCAUGCGNNNNNNGUAANNNNNNNGGUGUGAAGCNNNNNNGACAAUGUANNNNNNNNNNNNNNNNNNNNGCUAGNNN
NNNNNNNUAUGGNNAGCGAAAGCGAAUNNNNGNNNNNNGGCGUNGUGAUCCUUNNNNNNNNNNNUUCGNNNNNNNNN
NNNNNNNNNNNNNNNNNNUCCCGCAACUGNNNNNNNNNGAACGAUCNNNNNNNNNNCNGCGUACANNNNGNNCCUAAGN
NNNNGAAUGAAAGUNNNNNNNNNNNNNGAACUUGGUAAGCCCAAUANNNNCCCUUUCGAGGAGGNNNNNNNGCAAN
NNNNNNUAUNNNNUAGUGGGUAAAAGACANNNNAGGAAGCAAAUGCNNNNNUGUAACGNNNNNGAUAGNNNCUGU
UGNNNUAANNUGAAAGNNUGCAGACUUNNNNUAGGGUGNNNNNNNNUUCGNNNNNNNNUGCUU
```

The intron Arq.I2 was generated using the command:

```
python aRNAque.py -g 300 --verbose --
target="......((((...(((((((((((((.((((.......))))((((((.........)))))).(((
((((((((((..(.((((((....)))))).)..))....)))))).........(((((((....)))))))..
)))))...((((((..((((.((....))...(((((........((((((...........((((((....)))
)))....((((((((((((((((.........)))))))))..........)))))))))))))...))))))))
)...))))))..........)))))))))))))..))))....(((((.(((((..(((....)))..(((((((
(....))))))))...))))).).......(((((((..(((.....((((((((...))))))))...((
(((....))))).((((....)))))))...)))))))...((((((((((....))))))))))..." --
C=GUGCGACAAGAAGUNNNNNNNNNNNNNUNNNNNUGAAAGUNNNNNNNNNNNNAAUCGUUCANNNNNNNNNNCUANNNN
NNCAUGCGNNNNNNGUAANNNNNNNGGUGUGAAGCNNNNNNGACAAUGUANNNNNNNNNNNNNNNNNNNNGCUAGNNN
NNNNNNNUAUGGNNAGCGAAAGCGAAUNNNNGNNNNNNGGCGUNGUGAUCCUUNNNNNNNNNNNUUCGNNNNNNNNN
NNNNNNNNNNNNNNNNNNUCCCGCAACUGNNNNNNNNNGAACGAUCNNNNNNNNNNCNGCGUACANNNNGNNCCUAAGN
NNNNGAAUGAAAGUNNNNNNNNNNNNNGAACUUGGUAAGCCCAAUANNNNCCCUUUCGAGGAGGNNNNNNNGCAAN
NNNNNNUAUNNNNUAGUGGGUAAAAGACANNNNAAAAAGCAAAUGCNNNNNUGUAAUGNNNNNGAUAGNNNCUGU
UGNNNUAANNUGAAAGNNUGCUGACUUNNNNUAGGGUGNNNNNNNNUUCGNNNNNNNNUGCUU
```

The intron Arq.I3 was generated using the command:

```
python aRNAque.py --verbose --
target="......((((...((((((((((((..((((.......))))((((((.........)))))).(((
((((((((((..(.((((((....)))))).)..))....)))))).........(((((((....)))))))..
)))))...((((((..((((.((....))...(((((........((((((...........((((((....)))
)))....((((((((((((((((.........)))))))))..........)))))))))))))...))))))))
)...))))))........))))))))))))..))))...(((((.(((((..(((....)))..((((((((...
.))))))))...))))).))))).......(((((((..(((.....((((((((...))))))))...((((..
...)))).((((....)))))))...)))))))...((((((....))))))" --
C=GUGCGACAAGAAGUNNNNNNNNNNNNAUNNNNNUGAAAUUNNNNNNNNNNNNAAUCGUUCANNNNNNNNNNCUANNNN
NNCAUGCGNNNNNNGUAANNNNNNNGGUGUGAAGCNNNNNNGACAAUGUANNNNNNNNNNNNNNNNNNNNGCUAGNNN
NNNNNNNUAUGGNNAGCGAAAGCGAAUNNNNGNNNNNNGGCUUCGUGAUCCUUNNNNNNNNNNNUUCGNNNNNNNNN
NNNNNNNNNNNNNNNNNNUCCCGCAACUGNNNNNNNNNGAACGAUCNNNNNNNNNNCGGAGUACANNNNGNNCCUAAGN
NNNNAUUAAGGNNNNNNNNNNNGAACUUGGUAAGCCCAAUANNNNCCCUUUCGAGGAGGNNNNNNNGCAANNNNNN
NNUAUNNNNUAGUGGGUAAAGGACANNNNAAAAAGCGAAUGCNNNNNUGUAAUGNNNNNGAUAGNNNNNAUANNN
UAANNUGAAAGNNUGCUGACUUNNNNUAGGGUGNNNUUCGNNNUGC -n 1000 -EDg 100 -g 300 -msf
100 --log
```

Molar fractions of RNA species from PAGE experiments

Raw band intensity data from scanned gels was extracted using the Azurespot image analysis software (Azure Biosystems). Analysis was not conducted on species lacking the intron (i.e. the ligated and unligated exons). The molar fraction of each molecular species was calculated in the following way:

$$F_i = \frac{I_i}{L_c} \cdot \frac{U_{max}}{U_i}$$

Where F_i is the molar fraction of the molecular species i, I_i is the band intensity assigned to species i, L_c is the sum of all corrected band intensities in the given lane, U_{max} is the number of Us present in the molecular species with the most Us among species in the lane, and U_i is the number of Us present in the species i. The values of I_i for each band are acquired from Azurespot (Azure Biosystems), while L_c is calculated in the following way:

$$L_c = \sum I_j \cdot \frac{U_{max}}{U_j}$$

Where I_j is the intensity of a given band assigned to species j in the lane, and U_j is the number of Us present in the species j.

Model fitting

The time course data was fit using version 4.44 of the COPASI biochemical system simulator.[1] To simplify the reaction model the intensities of all bands belonging to species that have undergone branching were summed and fitted as one species, thus the second step of splicing is not modeled. The experimental datapoints representing the linear intron band were modeled to be the sum of the product of hydrolytic splicing, and broken lariat molecules which possess an identical electrophoretic mobility under our conditions. We understood the two reaction phases of precursor depletion, which we observed under all conditions tested, to be reflective of different processes being rate limiting at different times. Our model assumes the existence of two populations of precursor molecules, one that is prefolded at t=0 in the conformation that positions D6 for the first step of splicing (the active precursor population, "PRE_active"), and an inactive population ("PRE_inactive") that finds itself in the conformation responsible for the second step of splicing at t=0, and must first undergo a slow and rate-limiting conformational change (k_{conf}) to the active conformation before it can branch. Thus, in the initial fast reaction phase, branching is rate limiting (which could reflect the rate of folding or catalysis), and in the second reaction phase, after the prefolded active precursors are depleted, the slow conformational change is rate limiting. The active precursor is converted to either the branched species ("BRANCH"), or the linear intron ("LIN"), through branching (k_{branch}) and hydrolysis ($k_{hydrolysis}$) reactions, respectively, that are both modelled as irreversible reactions (main text, figure 2B). Lastly, the branched species are modeled to undergo an irreversible "breaking" reaction (k_{break}), intended to represent the cleavage of covalent backbone bonds, breaking the circular intron lariat ("LIN_dead"), and causing it to comigrate with the product of hydrolytic splicing — the linear intron (main text, figure 2B).

The following is a description of the model in the Antimony notation:

```
// Reactions:
branching: PRE_active => BRANCH; branching_k1*PRE_active;

hydrolysis: PRE_active => LIN; hydrolysis_k1*PRE_active;
break: BRANCH => LIN_dead; break_k1*BRANCH;
conf: PRE_inactive => PRE_active; conf_k1*PRE_inactive;

// Assignment Rules:
PRE_total := PRE_inactive + PRE_active;
LIN_total := LIN + LIN_dead;

// Species initializations:
PRE_active = 0;
LIN = 0;
BRANCH = 0;
LIN_dead = 0;
PRE_inactive = 100 - PRE_active;
```

Within the same temperature group, as many parameters as possible were constrained to be of an identical value across all Mg^{2+} concentrations. This resulted in all parameters, except the percentage of active precursor at t=0 to be the same value in the 37°C experiments (Table S3).

Statistics

The 95% confidence intervals (95% CIs) for each fitted parameter were determined according to Schaber 2012,[2] according to which the 95% confidence region of a parameter can be calculated with the help of the following equation:

$$\left\{p : SSR(p) \leq SSR(\hat{p}) \cdot \left(1 + \frac{m}{n-m} \cdot F^{\alpha}_{m,n-m}\right)\right\}$$

Which describes a set of all parameters p such that its sum of square residuals $SSR(p)$ is less than or equal to the minimal SSR achieved with the best fit parameters $\hat{p}$ multiplied by the term on the right. Here m is the number of parameters, n is the number of data points, and $F^{\alpha}_{m,n-m}$ is the α-quantile of the of the F-Ratio distribution, where $\alpha = 0.05$. This term on the right is calculated for a fitted model, and its value recorded. Afterwards, a series of regressions is performed on the dataset across a series of p values for each parameter, and the SSR of each regression corresponding to the value of p is recorded. The values of p where $SSR(p)$ equals to $SSR(\hat{p})$ multiplied by the term on the right are interpolated, and these values are taken to be the lower and upper bounds of the parameter p in the 95% CI.

Alignments

Intron sequences were aligned by hand with the wild-type intron P.li.LSU.I2, in order to ensure that the alignment was based on group II intron structural domains, rather than primary sequence, which may result in misalignment. The alignments generated this way were used as an input for the "alignment_percent_identity.py" script (Supplementary Data 2). The output generated by the script is also provided as "alignment_results.fasta" (Supplementary Data 2), and the pairwise sequence identities are reported inTable S22.

Image processing using ImageJ

The relative fluorescence intensity values of individual colonies on agar plates were extracted using ImageJ. Images were converted to 8-bit and the threshold was adjusted to highlight colonies on the plate of interest, resulting in a binary image. Watershed was applied to separate colonies. Particles were analysed to produce corresponding regions of interest (ROIs) for each colony. The grayscale image was then re-imported into ImageJ and the area and mean signal intensity of each colony was measured using the ROIs and the grayscale image. Before normalization, noise signals resulting from thresholding in ImageJ were removed from all data sets. The data of each replicate was processed individually. To correct signal intensities, first background fluorescence stemming from agar plates was subtracted and values normalized by area:

$$I_{c_i} = \frac{(I_{r_i} - I_b)}{A_i}$$

Where I_{c_i} is the corrected signal intensity of colony i, I_{r_i} is the raw signal intensity extracted from ImageJ, I_b is the signal intensity of the agar plate and A_i is the area of colony i. Signal intensities were then further normalized relative to the median of signal intensity of colonies on glucose plates (repressed expression):

$$I_{n_i} = \frac{I_{c_i}}{\tilde{x}(I_{c_{glu}})}$$

Where I_{n_i} is the normalized signal intensity of colony i and $\tilde{x}\left(I_{c_{glu}}\right)$ is the median of signal intensity of colonies on glucose plates of the respective replicate. To determine statistical significance of differences in signal intensities between samples, a one-sided Mann–Whitney-U-Test using the ratios between the median fluorescence of colonies on arabinose plates (induced) and median fluorescence of colonies on glucose plates (repressed) of each respective replicate was performed.

Trypsin digest of PURExpress reactions

Trypsin digestions of PURExpress reactions were performed as described by Doerr *et al.*[3] 7.5 µL of PURExpress reactions were combined with 15 µL of 100 mM Tris-HCl pH 8.0, 1.5 µL 20 mM $CaCl_2$ and 4.85 µL of ddH_2O. Samples were incubated at 90 °C for 10 min and left to cool to room temperature, after which 1.1 µL of 1 mg/mL Trypsin-ultra™, Mass Spectrometry Grade (NEB) were added to samples. Samples were incubated overnight at 37 °C, after which 3 µL of 10% trifluoroacetic aced (TFA) were added to samples. Samples were centrifuged in table-top centrifuge at 16,000 g for 10 min and supernatant was transferred to clean tubes. Samples were flash-frozen and stored at –80 °C. Purified sfGFP, which was used as a standard, was digested using Trypsin-ultra™, Mass Spectrometry Grade (NEB) in a 40:1 ratio (sfGFP:trypsin) according to the manufacturer's instructions. A 20 µL reaction containing, 2 µg of sfGFP (100 ng/µL), 1x of 2x Trypsin-ultra reaction buffer, 2.5 ng/µL of Trypsin was incubated at 37 °C for two hours, after which 1% formic acid was added to a final concentration of 0.1% of formic acid.

Proteomic analysis

Before proteomic analysis, a virtual trypsin digest of proteins in PURExpress and sfGFP were performed using Skyline,[4] to identify eight individual peptides of sfGFP not found in PURExpress. Mass spectrometric analysis of tryptic peptides was performed on an ACQUITY Premier LC system coupled to a Xevo TQ Absolute Triple Quadrupole Mass Spectrometry system using MassLynx mass spectrometry software (Waters Corporation, USA). From the trypsin digested samples, 10 µL were injected onto an ACQUITYTM Premier Peptide CSH C18 column (Waters Corporation, USA). Peptides were separated in a gradient of buffer A1 (1% formic acid in Milli-Q) and buffer B1 (1% formic acid in acetonitrile) at a flow rate of 300 µL per minute at a column temperature of 40°C. The column was equilibrated with a 98:2 ratio of buffer A1 to B1. After 15 min, the ratio was changed to 0:100 buffer A1 to B1 in 10 s and held for another 20 min. Finally, the column was flushed with 98:2 buffer A1 to B1 for 5 min. Supplementary Table S5Table S6 show the transitions of the measurements observed in each experiment.

Supplementary Figures

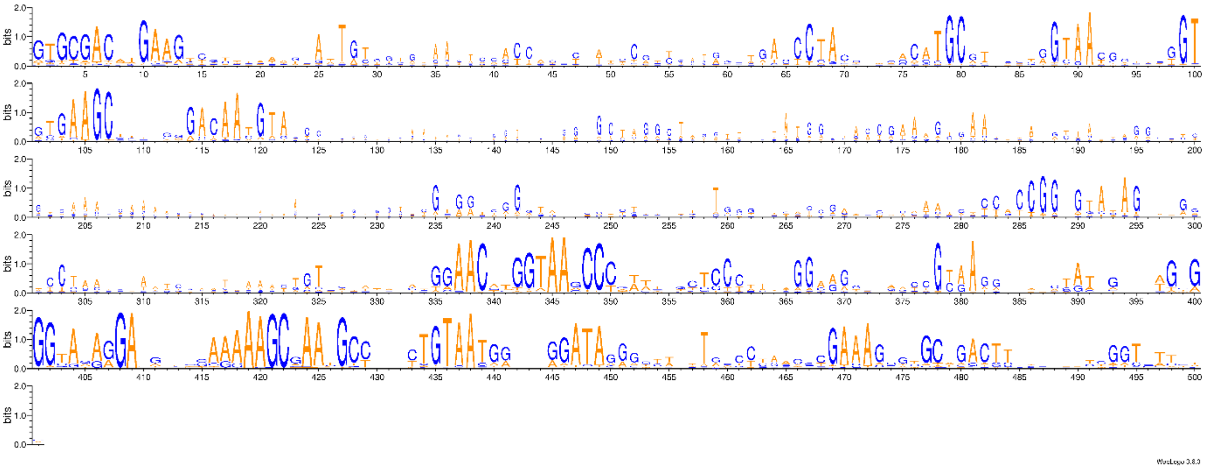

Figure S1: Consensus logo generated by rMSA[5] from the multiple-sequence alignment of the first three domains of the intron P.li.LSU.I2.

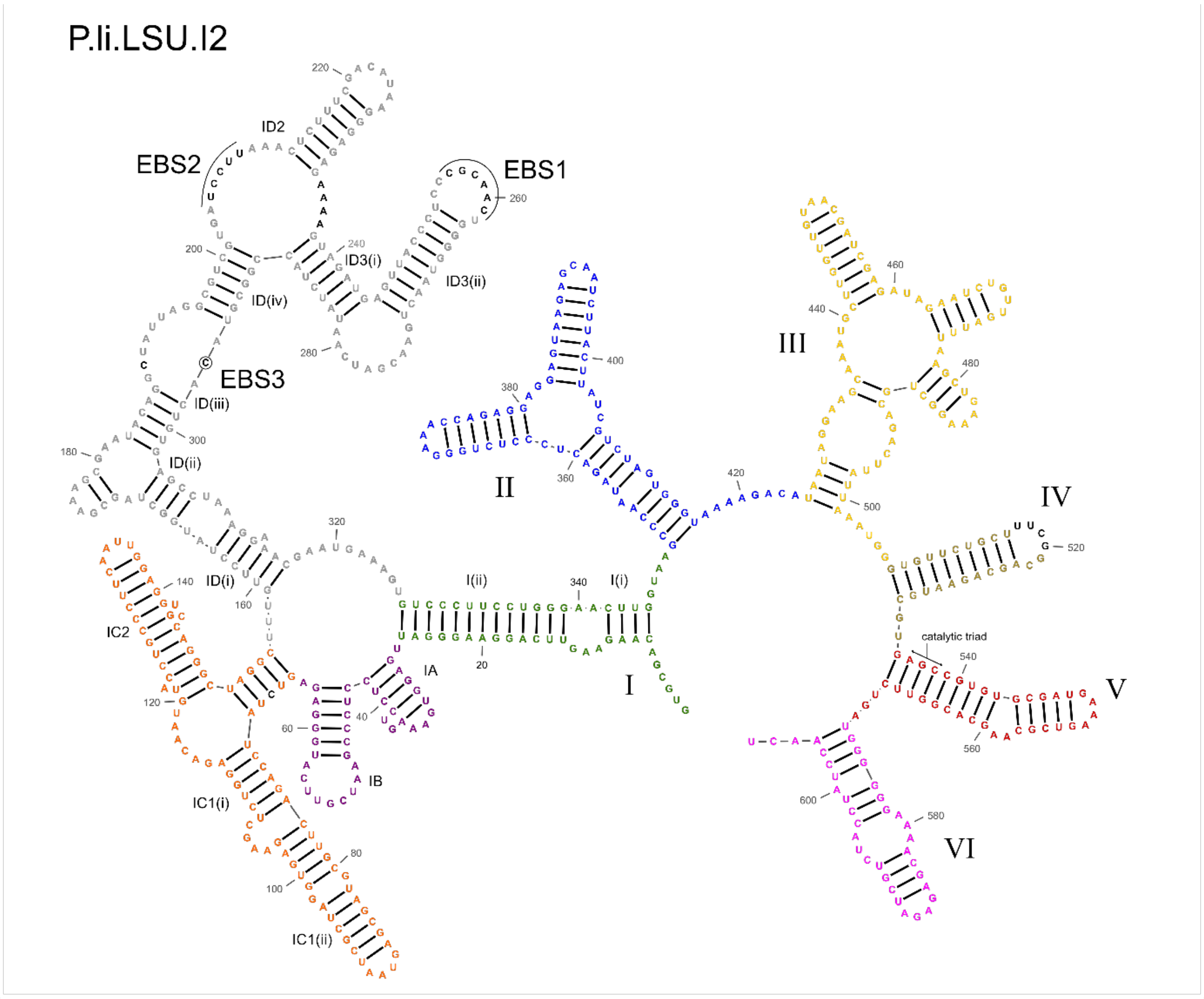

Figure S2: Secondary structure diagram of P.li.LSU.I2.[6] Mutated regions are in black font. The different intron domains are indicated by colour. Exon bindings sites (EBS) 1-3 bind to respective intron binding sites (IBS) on the exons.

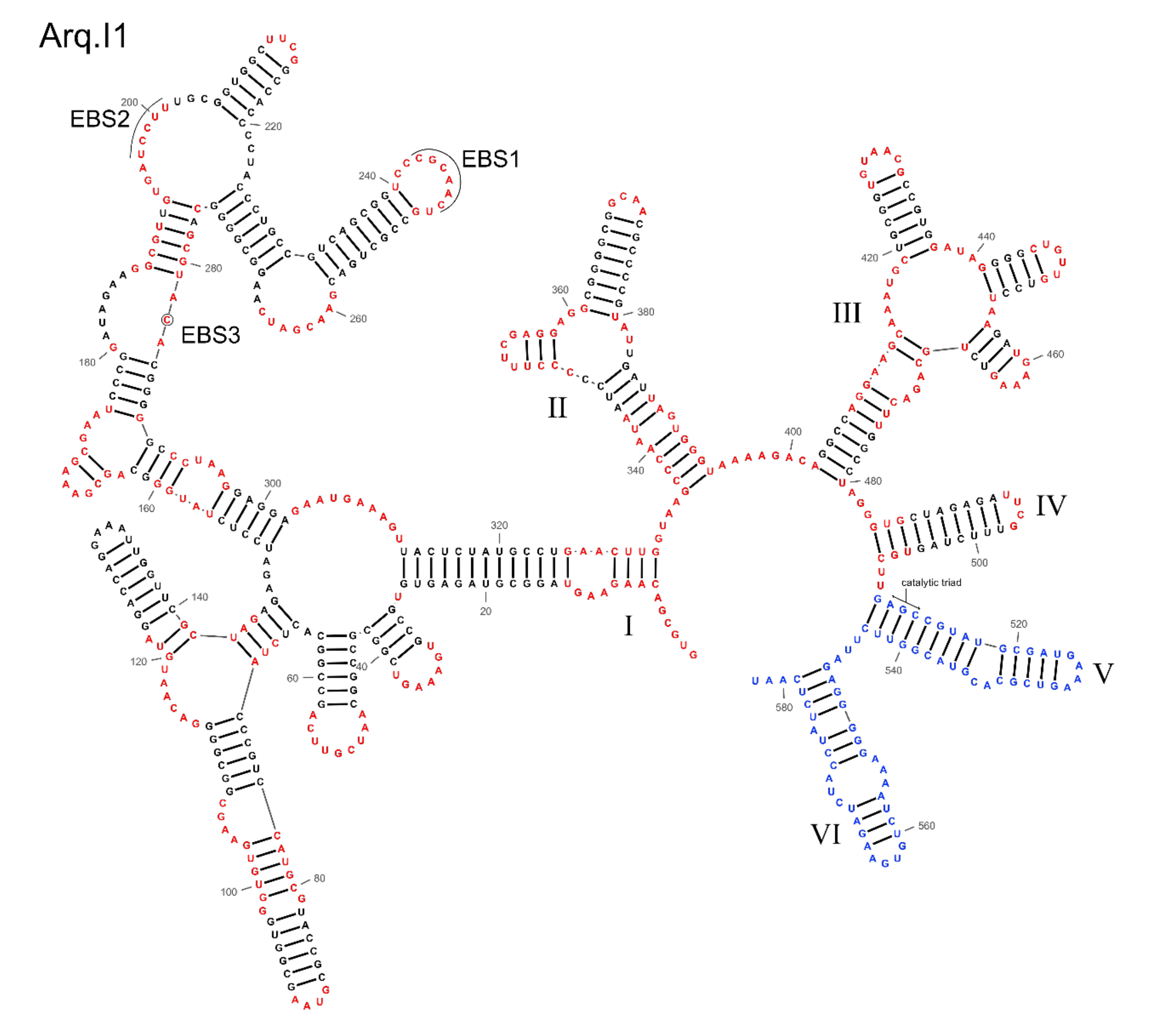

Figure S3: Secondary structure diagram of Arq.I1. Sequences which were constrained during inverse folding are highlighted in red, sequence regions generated by aRNAque are shown in black and rationally designed domains V and VI are highlighted in blue. Exon bindings sites (EBS) 1-3 bind to respective intron binding sites (IBS) on the exons.

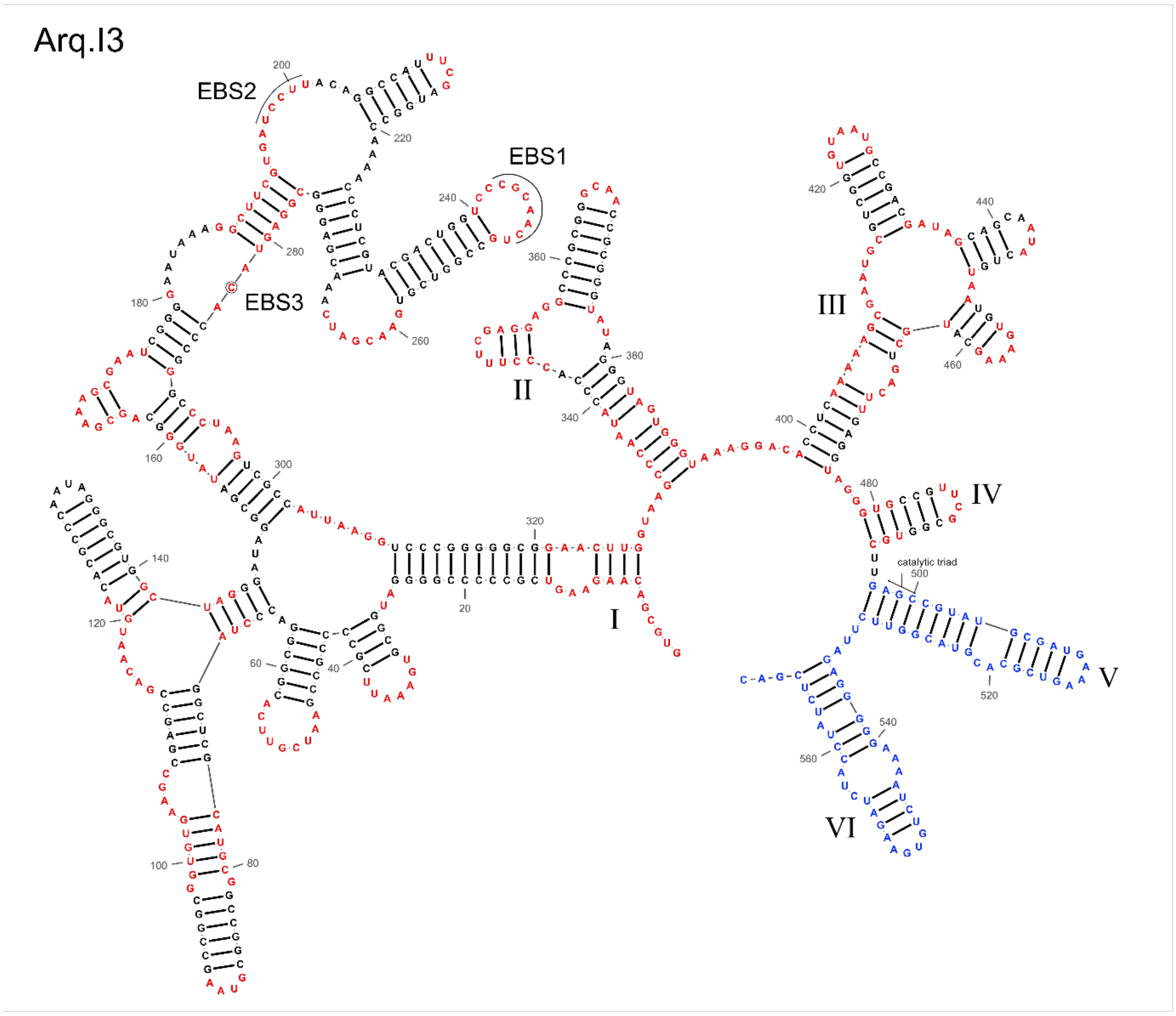

Figure S4: Secondary structure diagram of Arq.I3. Sequences which were constrained during inverse folding are highlighted in red, sequence regions generated by aRNAque are shown in black and rationally designed domains V and VI are highlighted in blue. Exon bindings sites (EBS) 1-3 bind to respective intron binding sites (IBS) on the exons.

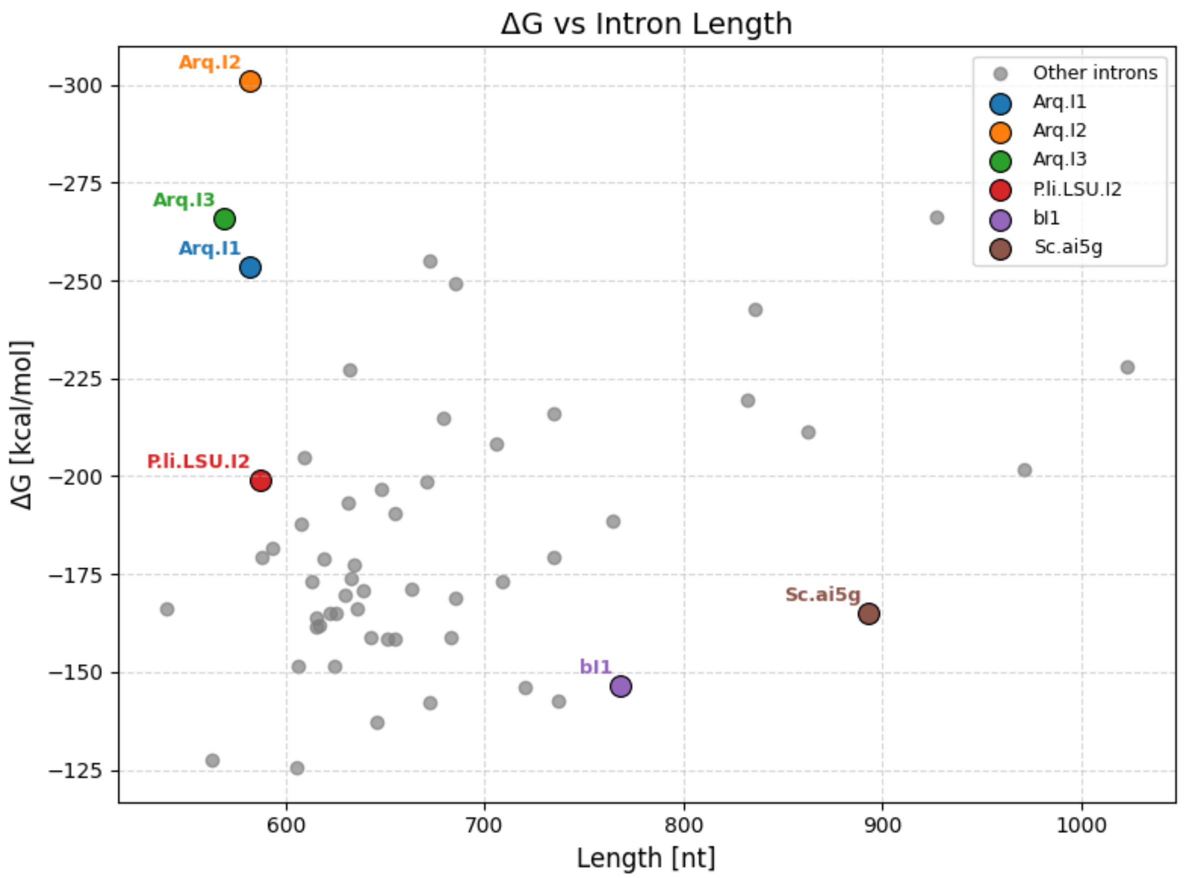

Figure S5: Predicted folding free energy (ΔG) vs group II intron length. The introns designed in this study, and especially Arq.I2 are predicted to have an extraordinarily stable secondary structure compared to naturally occurring group II introns.

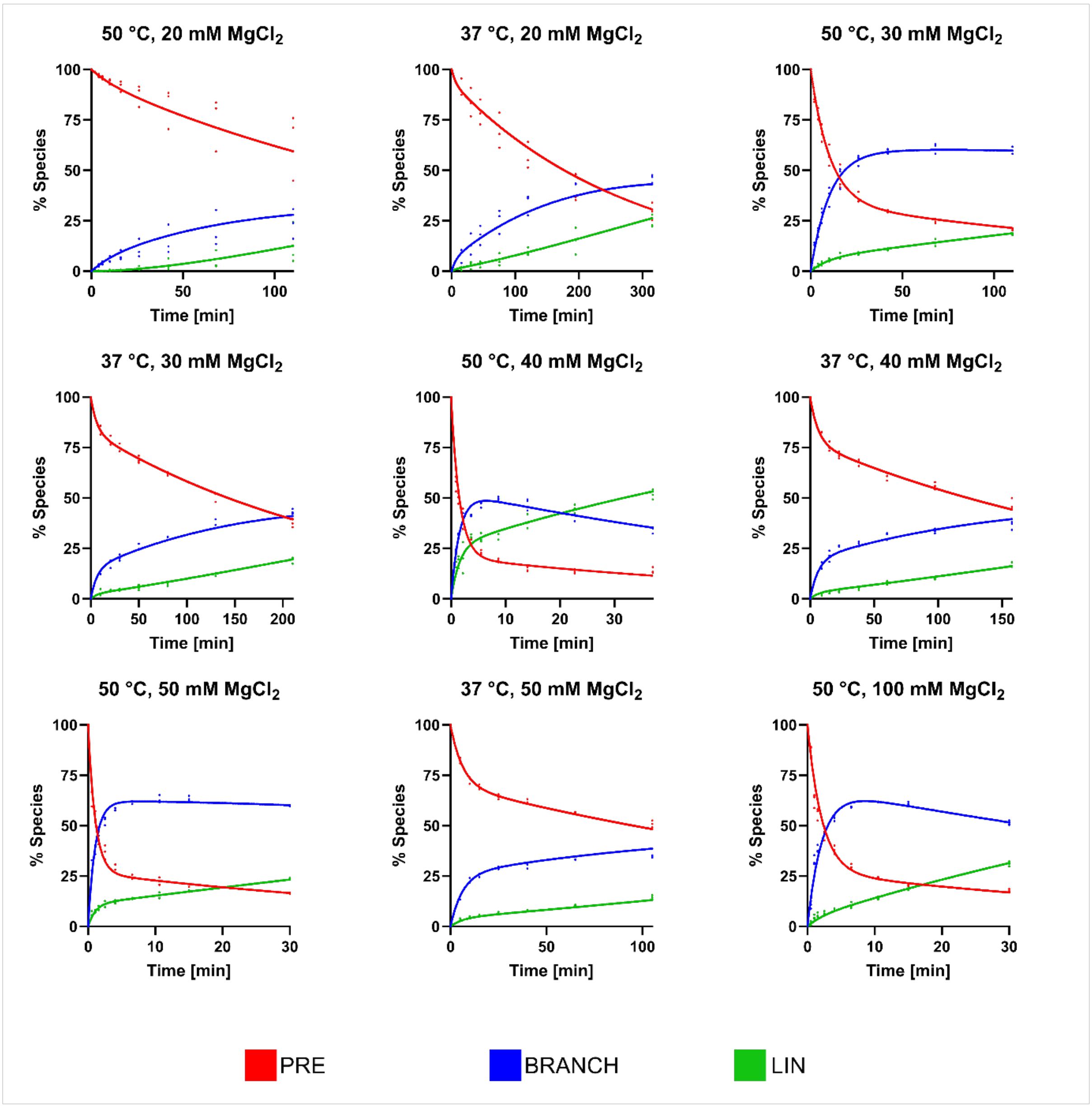

Figure S6: Experimental data (dots) of time courses alongside the kinetic model (lines). Reaction conditions are indicated by headers.

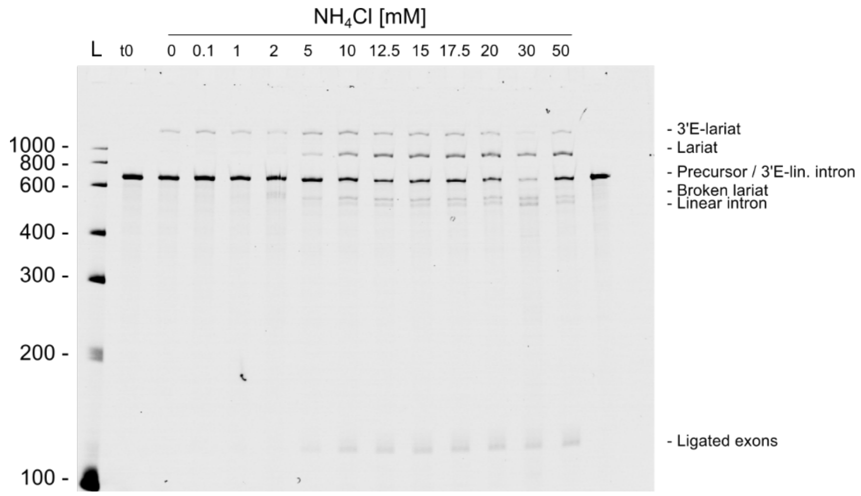

Figure S7: 4% Urea-PAGE of 0 - 50 mM NH_4Cl screen. Each sample contained 1.5 µM Arq.I2, 40 mM Tris HCl pH 7.5, 0.05 % v/v Tween 20, 25 mM $MgCl_2$, and varying concentrations of NH_4Cl as indicated. Samples were incubated at 45 °C for 1 hour.

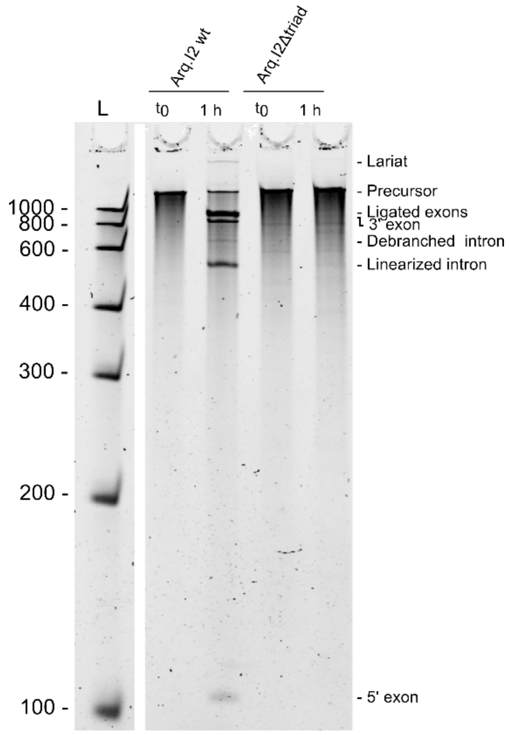

Figure S8: SYBR Gold stained 5% Urea PAGE of the splicing products from the linear Arq.I2-sfGFP in comparison to the Arq.I2Δtriad construct. The different product and intermediate species are annotated.

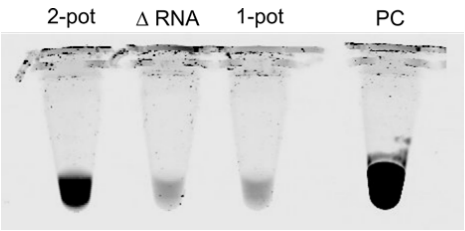

Figure S9: PURExpress reaction tubes scanned at 488/520 nm after six hours of incubation at 37°C.

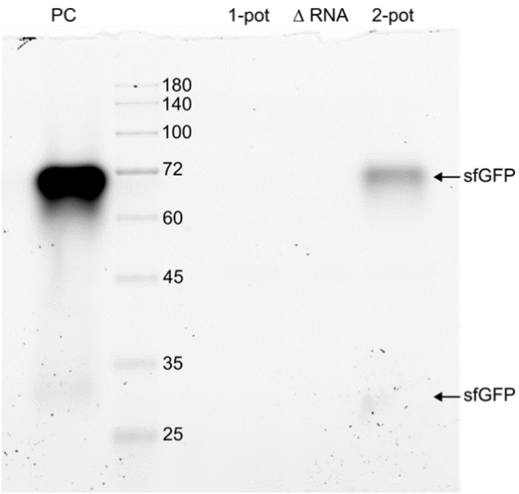

Figure S10: 10% SDS-PAGE of PURExpress reactions after six hours of incubation at 37°C. The gel was scanned at 488/520 nm to visualize sfGFP. Prior to loading, samples were heat denatured at 60°C for 10 minutes to avoid degradation of the fluorophore. This temperature was not sufficient to fully denature the protein, therefore most of the sfGFP appears as a band at approximately twice the expected size and only a small fraction at the expected size (26.8 kDa).[7]

MSKDCCGEELFTGVVPILVELDGDVNGHKFSVR**GEGEGDATNGK**LTLKFICTTGK
LPVPWPTLVTTLTYGVQCFSRYPDHMKRHDFFK**SAMPEGYVQER**TISFKDDGT
YKTRAEVK**FEGDTLVNR**IELKGIDFK**EDGNILGHKLEYNFNSHNVYITADK**QKN
GIKANFKIRHNVEDGSVQLADHYQQNTPIGDGPVLLPDNHYLSTQSVLSKDPNEKR
DHMVLLEFVTAAGITHGMDELYK*

Figure S11: Location of the tryptic sfGFP peptides along the sfGFP sequence. Detected peptides are highlighted in bold and either green or yellow.

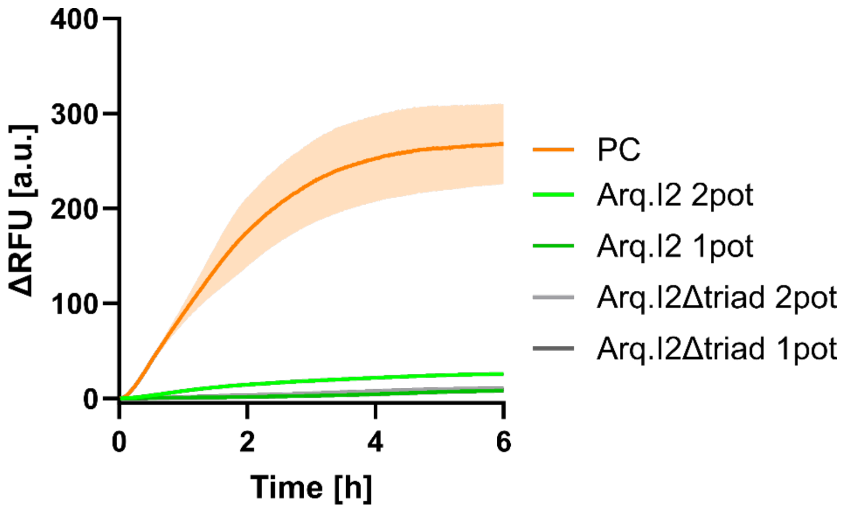

Figure S12: Relative fluorescence at 520 nm of the PURExpress real-time kinetics of sfGFP expression from RNA constructs (350 nM) containing either the coding sequence of sfGFP and Arq.I2 or Arq.I2Δtriad. A positive control using the expected post-splicing RNA sequence is shown in orange.

Supplementary Tables

Table S1: Sequences of designs, primers, transcripts and plasmids used. Lowercase symbolizes overhangs.

Name	Sequence
D56-Arq.I1-Arq.I2	GAGCCGUAUGCGAUGAAAGUCGCACGUACGGUUCUUAGAGGGGG AAAAUCUGUGAAGAUCUACCUAUCUCAAU
D56-Arq-I3	GAGCCGUAUGCGAUGAAAGUCGCACGUACGGUUCUUAGAGGGGG AAAAUCUGUGAAGAUCUACCUAUCUCGAC
Pr01-FWD-Pli	GCGAAATTAATACGACTCACTATAGGGAA
Pr02-REV-Pli-ArqI1[a]	mUmAGGTGACACTATAGAAGTGTGTATCG
Pr03-FWD-ArqI1	TCAGGGTTATTGTCTCATGAGCG
Pr04-FWD-ArqI2-ArqI3	GCGTAATACGACTCACTATAGGGATAATC
Pr05-REV-ArqI2-ArqI3	AGCGGATAACAATTTCACACAGG
Pr06-FWD-ArqI2sfGFP-gblock-assembly	CACTGGAGTTGTCCCAATTCTTG
Pr07-REV-ArqI2sfGFP-gblock-assembly	GCGTCAGGTAGGATCCGCTAATC
Pr08-FWD-ArqI2sfGFP-PC-assembly	tataccatggcacatATGAGCAAGGACTGTTGC
Pr09-REV-ArqI2sfGFP-PC-assembly	tctccttcttaaagttaaacaaaCCCAAAAAAACGGGTATGG
Pr10-FWD-ArqI2sfGFP-T7-PURE-transcript	ttgtaatacgactcactatagGGATCCTACCTGACGCT
Pr11-REV-ArqI2sfGFP-PURE-transcript	CTGCCGCCAGGCAAATTC
Pr12-FWD-Arq.I2Δtriad-NC-assembly	AGTCGCACGTACGGTTCTTAGAGG
Pr13-REV-Arq.I2Δtriad-NC-assembly	TTCATCGCATACGatcCAAGCACC
Arq.I2 + Exons[b]	GGGATAATCTAAGGACTGTTGCGGTGCGACAAGAAGTACCGCGG GCCCCTCGGCTGAAAGTGCCGGGGGCCAATCGTTCAGGCCCCAC ACTAGAGCCCCATGCGGGGGCCGTAAGGCCCCGGTGTGAAGCGG GCTCGACAATGTAGGCGCCCAAATGGGCGCCGCTAGTGAAACCC TGTATGGTGAGCGAAAGCGAATCCCAGAAAAAAGGCGTCGTGAT CCTTAAAGGAGCCTTCGGGCTCCAATACAGCGCGTACCTAGGTC CCGCAACTGCCTGGGTGGAACGATCATCGCGCTGCGGCGTACAT GGGGCACCTAAGCAGGGGAATGAAAGTGGGGCCCGCGGTGAACT

	TGGTAAGCCCAATAAGGACCCTTTCGAGGAGGCCGCCCGGCAAC<br>GGGCGGTATACCTTAGTGGGTAAAAGACACGCCAAAAAGCAAAT<br>GCCCCGCTGTAATGGCGGGGATAGGCCCTGTTGGGCTAAGGTGA<br>AAGCCTGCTGACTTGGCGTAGGGTGCCCCCGGTTCGCCGGGGGT<br>GCTTGAGCCGTATGCGATGAAAGTCGCACGTACGGTTCTTAGAG<br>GGGGAAAATCTGTGAAGATCTACCTATCTCAATGCCGGTGCGCA<br>TAACCACCTCAGTGCGAGCAAGGAAATCAGTTCTGGACCAGCGA<br>GCTGTGCTGCGACTCGTGGCGTAATCATGGTCATAGCTGTTTCC<br>TGTGTGAAATTGTTATCCGCT
gBlock for Arq.I2-sfGFP assembly [b]	TCCATAAGATTAGCGGATCCTACCTGACGCTTTTTATCGCAACT<br>CTCTACTGTTTCTCCATACCCGTTTTTTTGGGTTTGTTTAACTT<br>TAAGAAGGAGATATACCATGGCACATATGAGCAAGGACTGTTGC<br>GGTGCGACAAGAAGTACCGCGGGCCCCTCGGCTGAAAGTGCCGG<br>GGGCCAATCGTTCAGGCCCCACACTAGAGCCCCATGCGGGGGCC<br>GTAAGGCCCCGGTGTGAAGCGGGCTCGACAATGTAGGCGCCCAA<br>ATGGGCGCCGCTAGTGAAACCCTGTATGGTGAGCGAAAGCGAAT<br>CCCAGAAAAAAGGCGTCGTGATCCTTAAAGGAGCCTTCGGGCTC<br>CAATACAGCGCGTACCTAGGTCCCGCAACTGCCTGGGTGGAACG<br>ATCATCGCGCTGCGGCGTACATGGGGCACCTAAGCAGGGGAATG<br>AAAGTGGGGCCCGCGGTGAACTTGGTAAGCCCAATAAGGACCCT<br>TTCGAGGAGGCCGCCCGGCAACGGGCGGTATACCTTAGTGGGTA<br>AAAGACACGCCAAAAAGCAAATGCCCCGCTGTAATGGCGGGGAT<br>AGGCCCTGTTGGGCTAAGGTGAAAGCCTGCTGACTTGGCGTAGG<br>GTGCCCCCGGTTCGCCGGGGGTGCTTGAGCCGTATGCGATGAAA<br>GTCGCACGTACGGTTCTTAGAGGGGGAAAATCTGTGAAGATCTA<br>CCTATCTCAATGAGAAGAACTTTTCACTGGAGTTGTCCCAATTC<br>TTGTTGAATT
Arq.I2-sfGFP-PURE-IVT-template [b]	TTGTAATACGACTCACTATAGGGATCCTACCTGACGCTTTTTAT<br>CGCAACTCTCTACTGTTTCTCCATACCCGTTTTTTTGGGTTTGT<br>TTAACTTTAAGAAGGAGATATACCATGGCACATATGAGCAAGGA<br>CTGTTGCGGTGCGACAAGAAGTACCGCGGGCCCCTCGGCTGAAA<br>GTGCCGGGGGCCAATCGTTCAGGCCCCACACTAGAGCCCCATGC<br>GGGGGCCGTAAGGCCCCGGTGTGAAGCGGGCTCGACAATGTAGG<br>CGCCCAAATGGGCGCCGCTAGTGAAACCCTGTATGGTGAGCGAA<br>AGCGAATCCCAGAAAAAAGGCGTCGTGATCCTTAAAGGAGCCTT<br>CGGGCTCCAATACAGCGCGTACCTAGGTCCCGCAACTGCCTGGG<br>TGGAACGATCATCGCGCTGCGGCGTACATGGGGCACCTAAGCAG<br>GGGAATGAAAGTGGGGCCCGCGGTGAACTTGGTAAGCCCAATAA<br>GGACCCTTTCGAGGAGGCCGCCCGGCAACGGGCGGTATACCTTA<br>GTGGGTAAAAGACACGCCAAAAAGCAAATGCCCCGCTGTAATGG<br>CGGGGATAGGCCCTGTTGGGCTAAGGTGAAAGCCTGCTGACTTG<br>GCGTAGGGTGCCCCCGGTTCGCCGGGGGTGCTTGAGCCGTATGC<br>GATGAAAGTCGCACGTACGGTTCTTAGAGGGGGAAAATCTGTGA<br>AGATCTACCTATCTCAATGAGAAGAACTTTTCACTGGAGTTGTC<br>CCAATTCTTGTTGAATTAGATGGTGATGTTAATGGGCACAAATT<br>TTCTGTCCGTGGAGAGGGTGAAGGTGATGCTACAAACGGAAAAC<br>TCACCCTTAAATTTATTTGCACTACTGGAAAACTACCTGTTCCA<br>TGGCCAACACTTGTCACTACTCTGACCTATGGTGTTCAATGCTT<br>TTCCCGTTATCCGGATCACATGAAACGGCATGACTTTTTCAAGA<br>GTGCCATGCCCGAAGGTTATGTACAGGAACGCACTATATCTTTC<br>AAAGATGACGGGACCTACAAGACGCGTGCTGAAGTCAAGTTTGA<br>AGGTGATACCCTTGTTAATCGTATCGAGTTAAAAGGTATTGATT

	TTAAAGAAGATGGAAACATTCTCGGACACAAACTCGAGTACAAC<br>TTTAACTCACACAATGTATACATCACGGCAGACAAACAAAAGAA<br>TGGAATCAAAGCTAACTTCAAAATTCGCCACAACGTTGAAGATG<br>GTTCCGTTCAACTAGCAGACCATTATCAACAAAATACTCCAATT<br>GGCGATGGCCCTGTCCTTTTACCAGACAACCATTACCTGTCGAC<br>ACAATCTGTCCTTTCGAAAGATCCCAACGAAAAGCGTGACCACA<br>TGGTCCTTCTTGAGTTTGTAACTGCTGCTGGGATTACACATGGC<br>ATGGATGAGCTCTACAAATAGCGGATCCGAATTCGAGCTCCGT<br>CGACAAGCTTGGCTGTTTTGGCGGATGAGAGAAGATTTTCAGCC<br>TGATACAGATTAAATCAGAACGCAGAAGCGGTCTGATAAAACAG<br>AATTTGCCTGGCGGCAG
Arq.I1 pMiniT	TAATACGACTCACTATAGGGAGACGCACATGCGGCCGCCTCGAG<br>AATTCTGACGTCTTAATTAATTATTATCAGAAGGACTGTTGCGG<br>TGCGACAAGAAGTAGGCGTAGAGTGTGCCGTGAAAGTCGGCGCC<br>GGCAATCGTTCAGCCGGCACTCTACCCGTCCATGCGTACCGCGT<br>AAGCGGTGGGTGTGAAGCGGCGGGGACAATGTAGGACCAGGAAA<br>TTGGTTCGCTAGAGAGATCCTCTATGGGCAGCGAAAGCGAATCC<br>CGGATAGAAGGCGTTGTGATCCTTTGCGGTGGCTTCGGCCACCC<br>CTACCCTGCCGTCAGCGGTCCCGCAACTGCCGCTGACGAACGAT<br>CAAGGCGGGGCAGCGTACACGGGGGCCCTAAGGAGGAGAATGAA<br>AGTTACTCTATGCCTGAACTTGGTAAGCCCAATAATCCCCCTTT<br>CGAGGAGGCGGGGCGGCAACGCCCCGTATTGATTAGTGGGTAAA<br>AGACAGGCCAGGAAGCAAATGCTGCGGTGTAACGCCGTGGATAG<br>GGGCTGTTGTCCTAAGATGAAAGTCTGCAGACTTGGCCTAGGGT<br>GCTAGAGATTCGTTTCTAGTGCTTGAGCCGTATGCGATGAAAGT<br>CGCACGTACGGTTCTTAGAGGGGGAAAATCTGTGAAGATCTACC<br>TATCTCAATGCCGGTGCGCATAACCACCTCAGTGCGAGCAAGGA<br>AATCATGGTTTTTACCTCCTGAATTCGGATCCCTCGAGCGATAC<br>ACACTTCTATAGTGTCACCTAAATGCGTTTAAACCTTCCTGCAG<br>GTGACGATTACCTAACAATCGGTCGATTCGTTTGATGTTATGTT<br>TTGTTCTCGCTTTGGTTGGCAGGTTACGGCCAAGTTCGGTAAGA<br>GTGAGAGTTTTACAGTCAAGTAATGCGTGGCAAGCCAACGTTAA<br>GCTGTTGAGTCGTTTTAAGTGTAATTCGGGGCAGAATTGGTAAA<br>GAGAGTCGTGTAAAATATCGAGTTCGCACATCTTGTTGTCTGAT<br>TATTGATTTTTCGCGAAACCATTTAATCATATGACAAGATGTGT<br>GTCCACCTTAACTTAATGATTTTTACCAAAATCATTAGGGGATT<br>CATCAGCGCTGAGTGTGTAAATTAATTTTTATGCCGCAGCGGGC<br>CAGCAATTCTCGTGAATCATCGCTTAAACGGCCTGATTTCTGAA<br>TAGCTATGCGTAAGCGGGTGTTGTCTAACATTCTGCGTTCCTCT<br>TTATCCTGTCTGAACCGGCTGCATTAATGAATCGGCCAACGCGC<br>GGGGAGAGGCGGTTTGCGTATTGGGCGCTCTTCCGCTTCCTCGC<br>TCACTGACTCGCTGCGCTCGGTCGTTCGGCTGCGGCGAGCGGTA<br>TCAGCTCACTCAAAGGCGGTAATACGGTTATCCACAGAATCAGG<br>GGATAACGCAGGAAAGAACATGTGAGCAAAAGGCCAGCAAAAGG<br>CCAGGAACCGTAAAAAGGCCGCATTGCTGGCGTTTTTCCATAGG<br>CTCCGCCCCCCTGACGAGCATCACAAAAATCGACGCTCAAGTCA<br>GAGGTGGCGAAACCCGACAGGACTATAAAGATACCAGGCGTTTC<br>CCCCTGGAAGCTCCCTCGTGCGCTCTCCTGTTCCGACCCTGCCG<br>CTTACCGGATACCTGTCCGCCTTTCTCCCTTCGGGAAGCGTGGC<br>GCTTTCTCATAGCTCACGCTGTAGGTATCTCAGTTCGGTGTAGG<br>TCGTTCGCTCCAAGCTGGGCTGTGTGCACGAACCCCCCGTTCAG<br>CCCGACCGCTGCGCCTTATCCGGTAACTATCGTCTTGAGTCCAA

	CCCGGTAAGACACGACTTATCGCCACTGGCAGCAGCCACTGGTA<br>ACAGGATTAGCAGAGCGAGGTATGTAGGCGGTGCTACAGAGTTC<br>TTGAAGTGGTGGCCTAACTACGGCTACACTAGAAGAACAGTATT<br>TGGTATCTGCGCTCTGCTGAAGCCAGTTACCTTCGGAAAAAGAG<br>TTGGTAGCTCTTGATCCGGCAAACAAACCACCGCTGGTAGCGGT<br>GGTTTTTTTGTTTGCAAGCAGCAGATTACGCGCAGAAAAAAAGG<br>ATCTCAAGAAGATCCTTTGATCTTTTCTACGGGGTCTGACGCTC<br>AGTGGAACGAAAACTCACGTTAAGGGATTTTGGTCATGAGATTA<br>TCAAAAAGGATCTTCACCTAGATCCTTTTAAATTAAAAATGAAG<br>TTTTAAATCAATCTAAAGTATATATGAGTAAACTTGGTCTGACA<br>GTTACCAATGCTTAATCAGTGAGGCACCTATCTCAGCGATCTGT<br>CTATTTCGTTCATCCATAGTTGCCTGACTCCCCGTCGTGTAGAT<br>AACTACGATACGGGAGGGCTTACCATCTGGCCCCAGTGCTGCAA<br>TGATACCGCGAGATCCACGCTCACCGGCTCCAGATTTATCAGCA<br>ATAAACCAGCCAGCCGGAAGGGCCGAGCGCAGAAGTGGTCCTGC<br>AACTTTATCCGCCTCCATCCAGTCTATTAATTGTTGCCGGGAAG<br>CTAGAGTAAGTAGTTCGCCAGTTAATAGTTTGCGCAACGTTGTT<br>GCCATTGCTACAGGCATCGTGGTGTCACGCTCGTCGTTTGGTAT<br>GGCTTCATTCAGCTCCGGTTCCCAACGATCAAGGCGAGTTACAT<br>GATCCCCCATGTTGTGCAAAAAAGCGGTTAGCTCCTTCGGTCCT<br>CCGATCGTTGTCAGAAGTAAGTTGGCCGCAGTGTTATCACTCAT<br>GGTTATGGCAGCACTGCATAATTCTCTTACTGTCATGCCATCCG<br>TAAGATGCTTTTCTGTGACTGGTGAGTACTCAACCAAGTCATTC<br>TGAGAATAGTGTATGCGGCGACCGAGTTGCTCTTGCCCGGCGTC<br>AATACGGGATAATACCGCGCCACATAGCAGAACTTTAAAAGTGC<br>TCATCATTGGAAAACGTTCTTCGGGGCGAAAACTCTCAAGGATC<br>TTACCGCTGTTGAGATCCAGTTCGATGTAACCCACTCGTGCACC<br>CAACTGATCTTCAGCATCTTTTACTTTCACCAGCGTTTCTGGGT<br>GAGCAAAAACAGGAAGGCAAAATGCCGCAAAAAAGGGAATAAGG<br>GCGACACGGAAATGTTGAATACTCATACTCTTCCTTTTTCAATA<br>TTATTGAAGCATTTATCAGGGTTATTGTCTCATGAGCGGATACA<br>TATTTGAATGTATTTAGAAAAATAAACAAATAGGGGTTCCGCCC<br>GCGAAAT
Arq.I2 pUCIDT [b]	TAATACGACTCACTATAGGGATAATCTAAGGACTGTTGCGGTGC<br>GACAAGAAGTACCGCGGGCCCCTCGGCTGAAAGTGCCGGGGGCC<br>AATCGTTCAGGCCCCACACTAGAGCCCCATGCGGGGGCCGTAAG<br>GCCCCGGTGTGAAGCGGGCTCGACAATGTAGGCGCCCAAATGGG<br>CGCCGCTAGTGAAACCCTGTATGGTGAGCGAAAGCGAATCCCAG<br>AAAAAAGGCGTCGTGATCCTTAAAGGAGCCTTCGGGCTCCAATA<br>CAGCGCGTACCTAGGTCCCGCAACTGCCTGGGTGGAACGATCAT<br>CGCGCTGCGGCGTACATGGGGCACCTAAGCAGGGGAATGAAAGT<br>GGGGCCCGCGGTGAACTTGGTAAGCCCAATAAGGACCCTTTCGA<br>GGAGGCCGCCCGGCAACGGGCGGTATACCTTAGTGGGTAAAAGA<br>CACGCCAAAAAGCAAATGCCCCGCTGTAATGGCGGGGATAGGCC<br>CTGTTGGGCTAAGGTGAAAGCCTGCTGACTTGGCGTAGGGTGCC<br>CCCGGTTCGCCGGGGGTGCTTGAGCCGTATGCGATGAAAGTCGC<br>ACGTACGGTTCTTAGAGGGGGAAAATCTGTGAAGATCTACCTAT<br>CTCAATGCCGGTGCGCATAACCACCTCAGTGCGAGCAAGGAAAT<br>CAGTTCTGGACCAGCGAGCTGTGCTGCGACTCGTGGCGTAATCA<br>TGGTCATAGCTGTTTCCTGTGTGAAATTGTTATCCGCTCACAAT<br>TCCACACAACATACGAGCCGGAAGCATAAAGTGTAAAGCCTGGG<br>GTGCCTAATGAGTGAGCTAACTCACATTAATTGCGTTGCGCTCA

	CTGCCCGCTTTCCAGTCGGGAAACCTGTCGTGCCAGCTGCATTA<br>ATGAATCGGCCAACGCGCGGGGAGAGGCGGTTTGCGTATTGGGC<br>GCTCTTCCGCTTCCTCGCTCACTGACTCGCTGCGCTCGGTCGTT<br>CGGCTGCGGCGAGCGGTATCAGCTCACTCAAAGGCGGTAATACG<br>GTTATCCACAGAATCAGGGGATAACGCAGGAAAGAACATGTGAG<br>CAAAAGGCCAGCAAAAGGCCAGGAACCGTAAAAAGGCCGCGTTG<br>CTGGCGTTTTTCCATAGGCTCCGCCCCCCTGACGAGCATCACAA<br>AAATCGACGCTCAAGTCAGAGGTGGCGAAACCCGACAGGACTAT<br>AAAGATACCAGGCGTTTCCCCCTGGAAGCTCCCTCGTGCGCTCT<br>CCTGTTCCGACCCTGTCGCTTACCGGATACCTGTCCGCCTTTCT<br>CCCTTCGGGAAGCGTGGCGCTTTCTCATAGCTCACGCTGTAGGT<br>ATCTCAGTTCGGTGTAGGTCGTTCGCTCCAAGCTGGGCTGTGTG<br>CACGAACCCCCCGTTCAGCCCGACCGCTGCGCCTTATCCGGTAA<br>CTATCGTCTTGAGTCCAACCCGGTAAGACACGACTTATCGCCAC<br>TGGCAGCAGCCACTGGTAACAGGATTAGCAGAGCGAGGTATGTA<br>GGCGGTGCTACAGAGTTCTTGAAGTGGTGGCCTAACTACGGCTA<br>CACTAGAAGAACAGTATTTGGTATCTGCGCTCTGCTGAAGCCAG<br>TTACCTTCGGAAAAAGAGTTGGTAGCTCTTGATCCGGCAAACAA<br>ACCACCGCTGGTAGCGGTGGTTTTTTTGTTTGCAAGCAGCAGAT<br>TACGCGCAGAAAAAAAGGATCTCAAGAAGATCCTTTGATCTTTT<br>CTACGGGGTCTGACGCTCAGTGGAACGAAAACTCACGTTAAGGG<br>ATTTTGGTCATGAGATTATCAAAAAGGATCTTCACCTAGATCCT<br>TTTAAATTAAAAATGAAGTTTTAAATCAATCTAAAGTATATATG<br>AGTAAACTTGGTCTGACAGTTACCAATGCTTAATCAGTGAGGCA<br>CCTATCTCAGCGATCTGTCTATTTCGTTCATCCATAGTTGCCTG<br>ACTCCCCGTCGTGTAGATAACTACGATACGGGAGGGCTTACCAT<br>CTGGCCCCAGTGCTGCAATGATACCGCGAGATCCACGCTCACCG<br>GCTCCAGATTTATCAGCAATAAACCAGCCAGCCGGAAGGGCCGA<br>GCGCAGAAGTGGTCCTGCAACTTTATCCGCCTCCATCCAGTCTA<br>TTAATTGTTGCCGGGAAGCTAGAGTAAGTAGTTCGCCAGTTAAT<br>AGTTTGCGCAACGTTGTTGCCATTGCTACAGGCATCGTGGTGTC<br>ACGCTCGTCGTTTGGTATGGCTTCATTCAGCTCCGGTTCCCAAC<br>GATCAAGGCGAGTTACATGATCCCCCATGTTGTGCAAAAAAGCG<br>GTTAGCTCCTTCGGTCCTCCGATCGTTGTCAGAAGTAAGTTGGC<br>CGCAGTGTTATCACTCATGGTTATGGCAGCACTGCATAATTCTC<br>TTACTGTCATGCCATCCGTAAGATGCTTTTCTGTGACTGGTGAG<br>TACTCAACCAAGTCATTCTGAGAATAGTGTATGCGGCGACCGAG<br>TTGCTCTTGCCCGGCGTCAATACGGGATAATACCGCGCCACATA<br>GCAGAACTTTAAAAGTGCTCATCATTGGAAAACGTTCTTCGGGG<br>CGAAAACTCTCAAGGATCTTACCGCTGTTGAGATCCAGTTCGAT<br>GTAACCCACTCGTGCACCCAACTGATCTTCAGCATCTTTTACTT<br>TCACCAGCGTTTCTGGGTGAGCAAAAACAGGAAGGCAAAATGCC<br>GCAAAAAAGGGAATAAGGGCGACACGGAAATGTTGAATACTCAT<br>ACTCTACCTTTTTCAATATTATTGAAGCATTTATCAGGGTTATT<br>GTCTCATGAGCGGATACATATTTGAATGTATTTAGAAAAATAAA<br>CAAATAGGGGTTCCGCGCACATTTCCCCGAAAAGTGCCACCTGA<br>CGTCTAAGAAACCATTATTATCATGACATTAACCTATAAAAATA<br>GGCGTATCACGAGGCCCTTTCATCTCGCGCGTTTCGGTGATGAC<br>GGTGAAAACCTCTGACACATGCAGCTCCCCTAGACGGTCACAGC<br>TTGTCTGTAAGCGGATGCCGGGAGCAGACAAGCCCGTCAGGGCG<br>CGTCAGCGGGTGTTGGCGGGTGTCGGGGCTGGCTTAACTATGCG<br>GCATCAGAGCAGATTGTACTGAGAGTGCACCAAATGCGGTGTGA

	AATACCGCACAGATGCGTAAGGAGAAAATACCGCATCAGGCGCC<br>ATTCGCCATTCAGGCTGCGCAACTGTTGGGAAGGGCGATCGGTG<br>CGGGCCTCATCGCTATTACGCCAGCTGGCGAAAGGGGGATGTGC<br>TGCAAGGCGATTAAGTTGGGTAACGCCAGGGTTTTCCCAGTCAC<br>GACGTTGTAAAACGACGGCCAGTGCAACGCGATGACGATGGATA<br>GCGATTCATCGATGAGCTGACCCGATCGCCGCCGCCGGAGGGTT<br>GCGTTTGAGACAGGCGACAGATGCG
Arq.I3 pUCIDT	TAATACGACTCACTATAGGGATAATCTAAGGACTGTTGCGGTGC<br>GACAAGAAGTCGCCCCCGGGGATGGCGTGAAATTCGCCCCGCCG<br>AATCGTTCACGGCGGACCCTAGGCTCGCATGCGGCCGGCGTAAG<br>CCGGCGGTGTGAAGCCGAGCCGACAATGTACACGCCCAATAGGG<br>CGTGGCTAGGGATAGGCGATATGGGCAGCGAAAGCGAATCGGGG<br>AATAAAGGCTTCGTGATCCTTACAGGCCATTTCGATGGCCAAAA<br>CCCTCGTACGACTGGTCCCGCAACTGCCGGTCGTGAACGATCAA<br>ACGAGGGCGGAGTACACCCGGGCCCTAAGTCGCCATTAAGGTCC<br>CGGGGGCGGAACTTGGTAAGCCCAATACCCACCCTTTCGAGGAG<br>GCCCGCGGGCAACCGCGGGTATAGGGTAGTGGGTAAAGGACACC<br>TCAAAAAGCGAATGCGTCGGTGTAATGCCGACGATAGCAGCAAT<br>ACTGTAATGTGAAAGCATGCTGACTTGAGGTAGGGTGCCGTTCG<br>CGGTGCTTGAGCCGTATGCGATGAAAGTCGCACGTACGGTTCTT<br>AGAGGGGGAAAATCTGTGAAGATCTACCTATCTCGACGCCGGTG<br>CGCATAACCACCTCAGTGCGAGCAAGGAAATCAGTTCTGGACCA<br>GCGAGCTGTGCTGCGACTCGTGGCGTAATCATGGTCATAGCTGT<br>TTCCTGTGTGAAATTGTTATCCGCTCACAATTCCACACAACATA<br>CGAGCCGGAAGCATAAAGTGTAAAGCCTGGGGTGCCTAATGAGT<br>GAGCTAACTCACATTAATTGCGTTGCGCTCACTGCCCGCTTTCC<br>AGTCGGGAAACCTGTCGTGCCAGCTGCATTAATGAATCGGCCAA<br>CGCGCGGGGAGAGGCGGTTTGCGTATTGGGCGCTCTTCCGCTTC<br>CTCGCTCACTGACTCGCTGCGCTCGGTCGTTCGGCTGCGGCGAG<br>CGGTATCAGCTCACTCAAAGGCGGTAATACGGTTATCCACAGAA<br>TCAGGGGATAACGCAGGAAAGAACATGTGAGCAAAAGGCCAGCA<br>AAAGGCCAGGAACCGTAAAAAGGCCGCGTTGCTGGCGTTTTTCC<br>ATAGGCTCCGCCCCCCTGACGAGCATCACAAAAATCGACGCTCA<br>AGTCAGAGGTGGCGAAACCCGACAGGACTATAAAGATACCAGGC<br>GTTTCCCCCTGGAAGCTCCCTCGTGCGCTCTCCTGTTCCGACCC<br>TGTCGCTTACCGGATACCTGTCCGCCTTTCTCCCTTCGGGAAGC<br>GTGGCGCTTTCTCATAGCTCACGCTGTAGGTATCTCAGTTCGGT<br>GTAGGTCGTTCGCTCCAAGCTGGGCTGTGTGCACGAACCCCCCG<br>TTCAGCCCGACCGCTGCGCCTTATCCGGTAACTATCGTCTTGAG<br>TCCAACCCGGTAAGACACGACTTATCGCCACTGGCAGCAGCCAC<br>TGGTAACAGGATTAGCAGAGCGAGGTATGTAGGCGGTGCTACAG<br>AGTTCTTGAAGTGGTGGCCTAACTACGGCTACACTAGAAGAACA<br>GTATTTGGTATCTGCGCTCTGCTGAAGCCAGTTACCTTCGGAAA<br>AAGAGTTGGTAGCTCTTGATCCGGCAAACAAACCACCGCTGGTA<br>GCGGTGGTTTTTTTGTTTGCAAGCAGCAGATTACGCGCAGAAAA<br>AAAGGATCTCAAGAAGATCCTTTGATCTTTTCTACGGGGTCTGA<br>CGCTCAGTGGAACGAAAACTCACGTTAAGGGATTTTGGTCATGA<br>GATTATCAAAAAGGATCTTCACCTAGATCCTTTTAAATTAAAAA<br>TGAAGTTTTAAATCAATCTAAAGTATATATGAGTAAACTTGGTC<br>TGACAGTTACCAATGCTTAATCAGTGAGGCACCTATCTCAGCGA<br>TCTGTCTATTTCGTTCATCCATAGTTGCCTGACTCCCCGTCGTG<br>TAGATAACTACGATACGGGAGGGCTTACCATCTGGCCCCAGTGC

	TGCAATGATACCGCGAGATCCACGCTCACCGGCTCCAGATTTAT<br>CAGCAATAAACCAGCCAGCCGGAAGGGCCGAGCGCAGAAGTGGT<br>CCTGCAACTTTATCCGCCTCCATCCAGTCTATTAATTGTTGCCG<br>GGAAGCTAGAGTAAGTAGTTCGCCAGTTAATAGTTTGCGCAACG<br>TTGTTGCCATTGCTACAGGCATCGTGGTGTCACGCTCGTCGTTT<br>GGTATGGCTTCATTCAGCTCCGGTTCCCAACGATCAAGGCGAGT<br>TACATGATCCCCCATGTTGTGCAAAAAAGCGGTTAGCTCCTTCG<br>GTCCTCCGATCGTTGTCAGAAGTAAGTTGGCCGCAGTGTTATCA<br>CTCATGGTTATGGCAGCACTGCATAATTCTCTTACTGTCATGCC<br>ATCCGTAAGATGCTTTTCTGTGACTGGTGAGTACTCAACCAAGT<br>CATTCTGAGAATAGTGTATGCGGCGACCGAGTTGCTCTTGCCCG<br>GCGTCAATACGGGATAATACCGCGCCACATAGCAGAACTTTAAA<br>AGTGCTCATCATTGGAAAACGTTCTTCGGGGCGAAAACTCTCAA<br>GGATCTTACCGCTGTTGAGATCCAGTTCGATGTAACCCACTCGT<br>GCACCCAACTGATCTTCAGCATCTTTTACTTTCACCAGCGTTTC<br>TGGGTGAGCAAAAACAGGAAGGCAAAATGCCGCAAAAAAGGGAA<br>TAAGGGCGACACGGAAATGTTGAATACTCATACTCTACCTTTTT<br>CAATATTATTGAAGCATTTATCAGGGTTATTGTCTCATGAGCGG<br>ATACATATTTGAATGTATTTAGAAAAATAAACAAATAGGGGTTC<br>CGCGCACATTTCCCCGAAAAGTGCCACCTGACGTCTAAGAAACC<br>ATTATTATCATGACATTAACCTATAAAAATAGGCGTATCACGAG<br>GCCCTTTCATCTCGCGCGTTTCGGTGATGACGGTGAAAACCTCT<br>GACACATGCAGCTCCCCTAGACGGTCACAGCTTGTCTGTAAGCG<br>GATGCCGGGAGCAGACAAGCCCGTCAGGGCGCGTCAGCGGGTGT<br>TGGCGGGTGTCGGGGCTGGCTTAACTATGCGGCATCAGAGCAGA<br>TTGTACTGAGAGTGCACCAAATGCGGTGTGAAATACCGCACAGA<br>TGCGTAAGGAGAAAATACCGCATCAGGCGCCATTCGCCATTCAG<br>GCTGCGCAACTGTTGGGAAGGGCGATCGGTGCGGGCCTCATCGC<br>TATTACGCCAGCTGGCGAAAGGGGGATGTGCTGCAAGGCGATTA<br>AGTTGGGTAACGCCAGGGTTTTCCCAGTCACGACGTTGTAAAAC<br>GACGGCCAGTGCAACGCGATGACGATGGATAGCGATTCATCGAT<br>GAGCTGACCCGATCGCCGCCGCCGGAGGGTTGCGTTTGAGACAG<br>GCGACAGATGCG
P.li.LSU.I2 pMiniT	TAATACGACTCACTATAGGGAAATACTAAGGACTGTTGCGGTGC<br>GACAAGAAGTTCAGGAAGGGATTGAGGTGAAAGTCCTCCTCCCG<br>AATCGTTCATGGGAGAGTCTATCCAGACTTGCGTAGCGAGTAAT<br>CGCTAGGTGAGAAGCTCTGGAGACAATGTACCTGCCCTTCAATT<br>GGAGGTGCCAGGGCTAGGCTTTGTTCCTATGGCTAGCGAAAGCG<br>AATACAGGcTATTAGGCGTCGTGATCCTTAAACTCTTTCGACAT<br>AAGGGAGAGaaaaGTAGATGAGTTACCCTCCCGCAACTGGGGTA<br>ACTGAACGATCAATATCTACCGGCGTACACTGTGAGCCTAAAGG<br>AAACGAATGAAAGTGTCCCTTCCTGGGAACTTGGTAAGCCCAAT<br>AGACTCCCTCTGGGAAACCAGAGGAGGAGTAAGAGCAATCTTAC<br>TTATCGTCTAGTGGGTAAAAGACATAATAGGAAGCAAATGCTTG<br>GTTGTAACGATCGAGATAGAATCTGTTGATTTAAGCTGAAAGGC<br>TGCAGACTTATTAAATGGGTGTTCTGCTTTCGGCAGCAGAATGC<br>GTGAGCCGTGTGCGATGAAAGTCGCAAGCACGGTTCTGATGGGG<br>GGAAAACGAGAGATCGTCTACCTATCCAACTGCCGGTGCGCATA<br>ACCACCTCAGTGCGAGCAAGGAAATCATGGTTTTTACCTCCTGA<br>ATTCGGATCCCTCGAGCGATACACACTTCTATAGTGTCACCTAA<br>ATGCGTTTAAACCTTCCTGCAGGTGACGATTACCTAACAATCGG<br>TCGATTCGTTTGATGTTATGTTTTGTTCTCGCTTTGGTTGGCAG

	GTTACGGCCAAGTTCGGTAAGAGTGAGAGTTTTACAGTCAAGTA<br>ATGCGTGGCAAGCCAACGTTAAGCTGTTGAGTCGTTTTAAGTGT<br>AATTCGGGGCAGAATTGGTAAAGAGAGTCGTGTAAAATATCGAG<br>TTCGCACATCTTGTTGTCTGATTATTGATTTTTCGCGAAACCAT<br>TTAATCATATGACAAGATGTGTGTCCACCTTAACTTAATGATTT<br>TTACCAAAATCATTAGGGGATTCATCAGCGCTGAGTGTGTAAAT<br>TAATTTTTATGCCGCAGCGGGCCAGCAATTCTCGTGAATCATCG<br>CTTAAACGGCCTGATTTCTGAATAGCTATGCGTAAGCGGGTGTT<br>GTCTAACATTCTGCGTTCCTCTTTATCCTGTCTGAACCGGCTGC<br>ATTAATGAATCGGCCAACGCGCGGGGAGAGGCGGTTTGCGTATT<br>GGGCGCTCTTCCGCTTCCTCGCTCACTGACTCGCTGCGCTCGGT<br>CGTTCGGCTGCGGCGAGCGGTATCAGCTCACTCAAAGGCGGTAA<br>TACGGTTATCCACAGAATCAGGGGATAACGCAGGAAAGAACATG<br>TGAGCAAAAGGCCAGCAAAAGGCCAGGAACCGTAAAAAGGCCGC<br>ATTGCTGGCGTTTTTCCATAGGCTCCGCCCCCCTGACGAGCATC<br>ACAAAAATCGACGCTCAAGTCAGAGGTGGCGAAACCCGACAGGA<br>CTATAAAGATACCAGGCGTTTCCCCCTGGAAGCTCCCTCGTGCG<br>CTCTCCTGTTCCGACCCTGCCGCTTACCGGATACCTGTCCGCCT<br>TTCTCCCTTCGGGAAGCGTGGCGCTTTCTCATAGCTCACGCTGT<br>AGGTATCTCAGTTCGGTGTAGGTCGTTCGCTCCAAGCTGGGCTG<br>TGTGCACGAACCCCCCGTTCAGCCCGACCGCTGCGCCTTATCCG<br>GTAACTATCGTCTTGAGTCCAACCCGGTAAGACACGACTTATCG<br>CCACTGGCAGCAGCCACTGGTAACAGGATTAGCAGAGCGAGGTA<br>TGTAGGCGGTGCTACAGAGTTCTTGAAGTGGTGGCCTAACTACG<br>GCTACACTAGAAGAACAGTATTTGGTATCTGCGCTCTGCTGAAG<br>CCAGTTACCTTCGGAAAAAGAGTTGGTAGCTCTTGATCCGGCAA<br>ACAAACCACCGCTGGTAGCGGTGGTTTTTTTGTTTGCAAGCAGC<br>AGATTACGCGCAGAAAAAAAGGATCTCAAGAAGATCCTTTGATC<br>TTTTCTACGGGGTCTGACGCTCAGTGGAACGAAAACTCACGTTA<br>AGGGATTTTGGTCATGAGATTATCAAAAAGGATCTTCACCTAGA<br>TCCTTTTAAATTAAAAATGAAGTTTTAAATCAATCTAAAGTATA<br>TATGAGTAAACTTGGTCTGACAGTTACCAATGCTTAATCAGTGA<br>GGCACCTATCTCAGCGATCTGTCTATTTCGTTCATCCATAGTTG<br>CCTGACTCCCCGTCGTGTAGATAACTACGATACGGGAGGGCTTA<br>CCATCTGGCCCCAGTGCTGCAATGATACCGCGAGATCCACGCTC<br>ACCGGCTCCAGATTTATCAGCAATAAACCAGCCAGCCGGAAGGG<br>CCGAGCGCAGAAGTGGTCCTGCAACTTTATCCGCCTCCATCCAG<br>TCTATTAATTGTTGCCGGGAAGCTAGAGTAAGTAGTTCGCCAGT<br>TAATAGTTTGCGCAACGTTGTTGCCATTGCTACAGGCATCGTGG<br>TGTCACGCTCGTCGTTTGGTATGGCTTCATTCAGCTCCGGTTCC<br>CAACGATCAAGGCGAGTTACATGATCCCCCATGTTGTGCAAAAA<br>AGCGGTTAGCTCCTTCGGTCCTCCGATCGTTGTCAGAAGTAAGT<br>TGGCCGCAGTGTTATCACTCATGGTTATGGCAGCACTGCATAAT<br>TCTCTTACTGTCATGCCATCCGTAAGATGCTTTTCTGTGACTGG<br>TGAGTACTCAACCAAGTCATTCTGAGAATAGTGTATGCGGCGAC<br>CGAGTTGCTCTTGCCCGGCGTCAATACGGGATAATACCGCGCCA<br>CATAGCAGAACTTTAAAAGTGCTCATCATTGGAAAACGTTCTTC<br>GGGGCGAAAACTCTCAAGGATCTTACCGCTGTTGAGATCCAGTT<br>CGATGTAACCCACTCGTGCACCCAACTGATCTTCAGCATCTTTT<br>ACTTTCACCAGCGTTTCTGGGTGAGCAAAAACAGGAAGGCAAAA<br>TGCCGCAAAAAAGGGAATAAGGGCGACACGGAAATGTTGAATAC<br>TCATACTCTTCCTTTTTCAATATTATTGAAGCATTTATCAGGGT

	TATTGTCTCATGAGCGGATACATATTTGAATGTATTTAGAAAAA<br>TAAACAAATAGGGGTTCCGCCCGCG
Arq.I2-sfGFP pBAD33 [b]	AAGAAACCAATTGTCCATATTGCATCAGACATTGCCGTCACTGC<br>GTCTTTTACTGGCTCTTCTCGCTAACCAAACCGGTAACCCCGCT<br>TATTAAAAGCATTCTGTAACAAAGCGGGACCAAAGCCATGACAA<br>AAACGCGTAACAAAAGTGTCTATAATCACGGCAGAAAAGTCCAC<br>ATTGATTATTTGCACGGCGTCACACTTTGCTATGCCATAGCATT<br>TTTATCCATAAGATTAGCGGATCCTACCTGACGCTTTTTATCGC<br>AACTCTCTACTGTTTCTCCATACCCGTTTTTTTGGGTTTGTTTA<br>ACTTTAAGAAGGAGATATACCATGGCACATATGAGCAAGGACTG<br>TTGCGGTGCGACAAGAAGTACCGCGGGCCCCTCGGCTGAAAGTG<br>CCGGGGGCCAATCGTTCAGGCCCCACACTAGAGCCCCATGCGGG<br>GGCCGTAAGGCCCCGGTGTGAAGCGGGCTCGACAATGTAGGCGC<br>CCAAATGGGCGCCGCTAGTGAAACCCTGTATGGTGAGCGAAAGC<br>GAATCCCAGAAAAAAGGCGTCGTGATCCTTAAAGGAGCCTTCGG<br>GCTCCAATACAGCGCGTACCTAGGTCCCGCAACTGCCTGGGTGG<br>AACGATCATCGCGCTGCGGCGTACATGGGGCACCTAAGCAGGGG<br>AATGAAAGTGGGGCCCGCGGTGAACTTGGTAAGCCCAATAAGGA<br>CCCTTTCGAGGAGGCCGCCCGGCAACGGGCGGTATACCTTAGTG<br>GGTAAAAGACACGCCAAAAAGCAAATGCCCCGCTGTAATGGCGG<br>GGATAGGCCCTGTTGGGCTAAGGTGAAAGCCTGCTGACTTGGCG<br>TAGGGTGCCCCCGGTTCGCCGGGGGTGCTTGAGCCGTATGCGAT<br>GAAAGTCGCACGTACGGTTCTTAGAGGGGGAAAATCTGTGAAGA<br>TCTACCTATCTCAATGAGAAGAACTTTTCACTGGAGTTGTCCCA<br>ATTCTTGTTGAATTAGATGGTGATGTTAATGGGCACAAATTTTC<br>TGTCCGTGGAGAGGGTGAAGGTGATGCTACAAACGGAAAACTCA<br>CCCTTAAATTTATTTGCACTACTGGAAAACTACCTGTTCCATGG<br>CCAACACTTGTCACTACTCTGACCTATGGTGTTCAATGCTTTTC<br>CCGTTATCCGGATCACATGAAACGGCATGACTTTTTCAAGAGTG<br>CCATGCCCGAAGGTTATGTACAGGAACGCACTATATCTTTCAAA<br>GATGACGGGACCTACAAGACGCGTGCTGAAGTCAAGTTTGAAGG<br>TGATACCCTTGTTAATCGTATCGAGTTAAAAGGTATTGATTTTA<br>AAGAAGATGGAAACATTCTCGGACACAAACTCGAGTACAACTTT<br>AACTCACACAATGTATACATCACGGCAGACAAACAAAAGAATGG<br>AATCAAAGCTAACTTCAAAATTCGCCACAACGTTGAAGATGGTT<br>CCGTTCAACTAGCAGACCATTATCAACAAAATACTCCAATTGGC<br>GATGGCCCTGTCCTTTTACCAGACAACCATTACCTGTCGACACA<br>ATCTGTCCTTTCGAAAGATCCCAACGAAAAGCGTGACCACATGG<br>TCCTTCTTGAGTTTGTAACTGCTGCTGGGATTACACATGGCATG<br>GATGAGCTCTACAAATAGCGGGATCCGAATTCGAGCTCCGTCGA<br>CAAGCTTGGCTGTTTTGGCGGATGAGAGAAGATTTTCAGCCTGA<br>TACAGATTAAATCAGAACGCAGAAGCGGTCTGATAAAACAGAAT<br>TTGCCTGGCGGCAGTAGCGCGGTGGTCCCACCTGACCCCATGCC<br>GAACTCAGAAGTGAAACGCCGTAGCGCCGATGGTAGTGTGGGGT<br>CTCCCCATGCGAGAGTAGGGAACTGCCAGGCATCAAATAAAACG<br>AAAGGCTCAGTCGAAAGACTGGGCCTTTCGTTTTATCTGTTGTT<br>TGTCGGTGAACGCTCTCCTGAGTAGGACAAATCCGCCGGGAGCG<br>GATTTGAACGTTGCGAAGCAACGGCCCGGAGGGTGGCGGGCAGG<br>ACGCCCGCCATAAACTGCCAGGCATCAAATTAAGCAGAAGGCCA<br>TCCTGACGGATGGCCTTTTTGCGTTTCTACAAACTCTTTTGTTT<br>ATTTTTCTAAATACATTCAAATATGTATCCGCTCATGAGACAAT<br>AACCCTGATAAATGCTTCAATAATATTGAAAAAGGAAGAGTATG

	AGTATTCAACATTTCCGTGTCGCCCTTATTCCCTTTTTTGCGGC<br>ATTTTGCCTTCCTGTTTTTGCTCACCCAGAAACGCTGGTGAAAG<br>TAAAAGATGCTGAAGATCAGTTGGGTGCAGCAAACTATTAACTG<br>GCGAACTACTTACTCTAGCTTCCCGGCAACAATTAATAGACTGG<br>ATGGAGGCGGATAAAGTTGCAGGACCACTTCTGCGCTCGGCCCT<br>TCCGGCTGGCTGGTTTATTGCTGATAAATCTGGAGCCGGTGAGC<br>GTGGGTCTCGCGGTATCATTGCAGCACTGGGGCCAGATGGTAAG<br>CCCTCCCGTATCGTAGTTATCTACACGACGGGGAGTCAGGCAAC<br>TATGGATGAACGAAATAGACAGATCGCTGAGATAGGTGCCTCAC<br>TGATTAAGCATTGGTAACTGTCAGACCAAGTTTACTCATATATA<br>CTTTAGATTGATTTACGCGCCCTGTAGCGGCGCATTAAGCGCGG<br>CGGGTGTGGTGGTTACGCGCAGCGTGACCGCTACACTTGCCAGC<br>GCCCTAGCGCCCGCTCCTTTCGCTTTCTTCCCTTCCTTTCTCGC<br>CACGTTCGCCGGCTTTCCCCGTCAAGCTCTAAATCGGGGGCTCC<br>CTTTAGGGTTCCGATTTAGTGCTTTACGGCACCTCGACCCCAAA<br>AAACTTGATTTGGGTGATGGTTCACGTAGTGGGCCATCGCCCTG<br>ATAGACGGTTTTTCGCCCTTTGACGTTGGAGTCCACGTTCTTTA<br>ATAGTGGACTCTTGTTCCAAACTTGAACAACACTCAACCCTATC<br>TCGGGCTATTCTTTTGATTTATAAGGGATTTTGCCGATTTCGGC<br>CTATTGGTTAAAAAATGAGCTGATTTAACAAAAATTTAACGCGA<br>ATTTTAACAAAATATTAACGTTTACAATTTAAAAGGATCTAGGT<br>GAAGATCCTTTTTGATAATCTCATGACCAAAATCCCTTAACGTG<br>AGTTTTCGTTCCACTGAGCGTCAGACCCCGTAGAAAAGATCAAA<br>GGATCTTCTTGAGATCCTTTTTTTCTGCGCGTAATCTGCTGCTT<br>GCAAACAAAAAAACCACCGCTACCAGCGGTGGTTTGTTTGCCGG<br>ATCAAGAGCTACCAACTCTTTTTCCGAAGGTAACTGGCTTCAGC<br>AGAGCGCAGATACCAAATACTGTCCTTCTAGTGTAGCCGTAGTT<br>AGGCCACCACTTCAAGAACTCTGTAGCACCGCCTACATACCTCG<br>CTCTGCTAATCCTGTTACCAGTCAGGCATTTGAGAAGCACACGG<br>TCACACTGCTTCCGGTAGTCAATAAACCGGTAAACCAGCAATAG<br>ACATAAGCGGCTATTTAACGACCCTGCCCTGAACCGACGACCGG<br>GTCGAATTTGCTTTCGAATTTCTGCCATTCATCCGCTTATTATC<br>ACTTATTCAGGCGTAGCACCAGGCGTTTAAGGGCACCAATAACT<br>GCCTTAAAAAAATTACGCCCCGCCCTGCCACTCATCGCAGTACT<br>GTTGTAATTCATTAAGCATTCTGCCGACATGGAAGCCATCACAG<br>ACGGCATGATGAACCTGAATCGCCAGCGGCATCAGCACCTTGTC<br>GCCTTGCGTATAATATTTGCCCATGGTGAAAACGGGGGCGAAGA<br>AGTTGTCCATATTGGCCACGTTTAAATCAAAACTGGTGAAACTC<br>ACCCAGGGATTGGCTGAGACGAAAAACATATTCTCAATAAACCC<br>TTTAGGGAAATAGGCCAGGTTTTCACCGTAACACGCCACATCTT<br>GCGAATATATGTGTAGAAACTGCCGGAAATCGTCGTGGTATTCA<br>CTCCAGAGCGATGAAAACGTTTCAGTTTGCTCATGGAAAACGGT<br>GTAACAAGGGTGAACACTATCCCATATCACCAGCTCACCGTCTT<br>TCATTGCCATACGGAATTCCGGATGAGCATTCATCAGGCGGGCA<br>AGAATGTGAATAAAGGCCGGATAAAACTTGTGCTTATTTTTCTT<br>TACGGTCTTTAAAAAGGCCGTAATATCCAGCTGAACGGTCTGGT<br>TATAGGTACATTGAGCAACTGACTGAAATGCCTCAAAATGTTCT<br>TTACGATGCCATTGGGATATATCAACGGTGGTATATCCAGTGAT<br>TTTTTTCTCCATTTTAGCTTCCTTAGCTCCTGAAAATCTCGATA<br>ACTCAAAAAATACGCCCGGTAGTGATCTTATTTCATTATGGTGA<br>AAGTTGGAACCTCTTACGTGCCGATCAACGTCTCATTTTCGCCA<br>AAAGTTGGCCCAGGGCTTCCCGGTATCAACAGGGACACCAGGAT

	TTATTTATTCTGCGAAGTGATCTTCCGTCACAGGTATTTATTCG<br>GCGCAAAGTGCGTCGGGTGATGCTGCCAACTTACTGATTTAGTG<br>TATGATGGTGTTTTTGAGGTGCTCCAGTGGCTTCTGTTTCTATC<br>AGCTGTCCCTCCTGTTCAGCTACTGACGGGGTGGTGCGTAACGG<br>CAAAAGCACCGCCGGACATCAGCGCTAGCGGAGTGTATACTGGC<br>TTACTATGTTGGCACTGATGAGGGTGTCAGTGAAGTGCTTCATG<br>TGGCAGGAGAAAAAAGGCTGCACCGGTGCGTCAGCAGAATATGT<br>GATACAGGATATATTCCGCTTCCTCGCTCACTGACTCGCTACGC<br>TCGGTCGTTCGACTGCGGCGAGCGGAAATGGCTTACGAACGGGG<br>CGGAGATTTCCTGGAAGATGCCAGGAAGATACTTAACAGGGAAG<br>TGAGAGGGCCGCGGCAAAGCCGTTTTTCCATAGGCTCCGCCCCC<br>CTGACAAGCATCACGAAATCTGACGCTCAAATCAGTGGTGGCGA<br>AACCCGACAGGACTATAAAGATACCAGGCGTTTCCCCCTGGCGG<br>CTCCCTCGTGCGCTCTCCTGTTCCTGCCTTTCGGTTTACCGGTG<br>TCATTCCGCTGTTATGGCCGCGTTTGTCTCATTCCACGCCTGAC<br>ACTCAGTTCCGGGTAGGCAGTTCGCTCCAAGCTGGACTGTATGC<br>ACGAACCCCCCGTTCAGTCCGACCGCTGCGCCTTATCCGGTAAC<br>TATCGTCTTGAGTCCAACCCGGAAAGACATGCAAAAGCACCACT<br>GGCAGCAGCCACTGGTAATTGATTTAGAGGAGTTAGTCTTGAAG<br>TCATGCGCCGGTTAAGGCTAAACTGAAAGGACAAGTTTTGGTGA<br>CTGCGCTCCTCCAAGCCAGTTACCTCGGTTCAAAGAGTTGGTAG<br>CTCAGAGAACCTTCGAAAAACCGCCCTGCAAGGCGGTTTTTTCG<br>TTTTCAGAGCAAGAGATTACGCGCAGACCAAAACGATCTCAAGA<br>AGATCATCTTATTAATCAGATAAAATATTTGCTCATGAGCCCGA<br>AGTGGCGAGCCCGATCTTCCCCATCGGTGATGTCGGCGATATAG<br>GCGCCAGCAACCGCACCTGTGGCGCCGGTGATGCCGGCCACGAT<br>GCGTCCGGCGTAGAGGATCTGCTCATGTTTGACAGCTTATCATC<br>GATGCATAATGTGCCTGTCAAATGGACGAAGCAGGGATTCTGCA<br>AACCCTATGCTACTCCGTCAAGCCGTCAATTGTCTGATTCGTTA<br>CCAATTATGACAACTTGACGGCTACATCATTCACTTTTTCTTCA<br>CAACCGGCACGGAACTCGCTCGGGCTGGCCCCGGTGCATTTTTT<br>AAATACCCGCGAGAAATAGAGTTGATCGTCAAAACCAACATTGC<br>GACCGACGGTGGCGATAGGCATCCGGGTGGTGCTCAAAAGCAGC<br>TTCGCCTGGCTGATACGTTGGTCCTCGCGCCAGCTTAAGACGCT<br>AATCCCTAACTGCTGGCGGAAAAGATGTGACAGACGCGACGGCG<br>ACAAGCAAACATGCTGTGCGACGCTGGCGATATCAAAATTGCTG<br>TCTGCCAGGTGATCGCTGATGTACTGACAAGCCTCGCGTACCCG<br>ATTATCCATCGGTGGATGGAGCGACTCGTTAATCGCTTCCATGC<br>GCCGCAGTAACAATTGCTCAAGCAGATTTATCGCCAGCAGCTCC<br>GAATAGCGCCCTTCCCCTTGCCCGGCGTTAATGATTTGCCCAAA<br>CAGGTCGCTGAAATGCGGCTGGTGCGCTTCATCCGGGCGAAAGA<br>ACCCCGTATTGGCAAATATTGACGGCCAGTTAAGCCATTCATGC<br>CAGTAGGCGCGCGGACGAAAGTAAACCCACTGGTGATACCATTC<br>GCGAGCCTCCGGATGACGACCGTAGTGATGAATCTCTCCTGGCG<br>GGAACAGCAAAATATCACCCGGTCGGCAAACAAATTCTCGTCCC<br>TGATTTTTCACCACCCCCTGACCGCGAATGGTGAGATTGAGAAT<br>ATAACCTTTCATTCCCAGCGGTCGGTCGATAAAAAAATCGAGAT<br>AACCGTTGGCCTCAATCGGCGTTAAACCCGCCACCAGATGGGCA<br>TTAAACGAGTATCCCGGCAGCAGGGGATCATTTTGCGCTTCAGC<br>CATACTTTTCATACTCCCGCCATTCAGAG

Arq.I2-sfGFP post-splicing positive control pBAD33	AAGAAACCAATTGTCCATATTGCATCAGACATTGCCGTCACTGC<br>GTCTTTTACTGGCTCTTCTCGCTAACCAAACCGGTAACCCCGCT<br>TATTAAAAGCATTCTGTAACAAAGCGGGACCAAAGCCATGACAA<br>AAACGCGTAACAAAAGTGTCTATAATCACGGCAGAAAAGTCCAC<br>ATTGATTATTTGCACGGCGTCACACTTTGCTATGCCATAGCATT<br>TTTATCCATAAGATTAGCGGATCCTACCTGACGCTTTTTATCGC<br>AACTCTCTACTGTTTCTCCATACCCGTTTTTTTGGGTTTGTTTA<br>ACTTTAAGAAGGAGATATACCATGGCACATATGAGCAAGGACTG<br>TTGCGGAGAAGAACTTTTCACTGGAGTTGTCCCAATTCTTGTTG<br>AATTAGATGGTGATGTTAATGGGCACAAATTTTCTGTCCGTGGA<br>GAGGGTGAAGGTGATGCTACAAACGGAAAACTCACCCTTAAATT<br>TATTTGCACTACTGGAAAACTACCTGTTCCATGGCCAACACTTG<br>TCACTACTCTGACCTATGGTGTTCAATGCTTTTCCCGTTATCCG<br>GATCACATGAAACGGCATGACTTTTTCAAGAGTGCCATGCCCGA<br>AGGTTATGTACAGGAACGCACTATATCTTTCAAAGATGACGGGA<br>CCTACAAGACGCGTGCTGAAGTCAAGTTTGAAGGTGATACCCTT<br>GTTAATCGTATCGAGTTAAAAGGTATTGATTTTAAAGAAGATGG<br>AAACATTCTCGGACACAAACTCGAGTACAACTTTAACTCACACA<br>ATGTATACATCACGGCAGACAAACAAAAGAATGGAATCAAAGCT<br>AACTTCAAAATTCGCCACAACGTTGAAGATGGTTCCGTTCAACT<br>AGCAGACCATTATCAACAAAATACTCCAATTGGCGATGGCCCTG<br>TCCTTTTACCAGACAACCATTACCTGTCGACACAATCTGTCCTT<br>TCGAAAGATCCCAACGAAAAGCGTGACCACATGGTCCTTCTTGA<br>GTTTGTAACTGCTGCTGGGATTACACATGGCATGGATGAGCTCT<br>ACAAATAGCGGGATCCGAATTCGAGCTCCGTCGACAAGCTTGGC<br>TGTTTTGGCGGATGAGAGAAGATTTTCAGCCTGATACAGATTAA<br>ATCAGAACGCAGAAGCGGTCTGATAAAACAGAATTTGCCTGGCG<br>GCAGTAGCGCGGTGGTCCCACCTGACCCCATGCCGAACTCAGAA<br>GTGAAACGCCGTAGCGCCGATGGTAGTGTGGGGTCTCCCCATGC<br>GAGAGTAGGGAACTGCCAGGCATCAAATAAAACGAAAGGCTCAG<br>TCGAAAGACTGGGCCTTTCGTTTTATCTGTTGTTTGTCGGTGAA<br>CGCTCTCCTGAGTAGGACAAATCCGCCGGGAGCGGATTTGAACG<br>TTGCGAAGCAACGGCCCGGAGGGTGGCGGGCAGGACGCCCGCCA<br>TAAACTGCCAGGCATCAAATTAAGCAGAAGGCCATCCTGACGGA<br>TGGCCTTTTTGCGTTTCTACAAACTCTTTTGTTTATTTTTCTAA<br>ATACATTCAAATATGTATCCGCTCATGAGACAATAACCCTGATA<br>AATGCTTCAATAATATTGAAAAAGGAAGAGTATGAGTATTCAAC<br>ATTTCCGTGTCGCCCTTATTCCCTTTTTTGCGGCATTTTGCCTT<br>CCTGTTTTTGCTCACCCAGAAACGCTGGTGAAAGTAAAAGATGC<br>TGAAGATCAGTTGGGTGCAGCAAACTATTAACTGGCGAACTACT<br>TACTCTAGCTTCCCGGCAACAATTAATAGACTGGATGGAGGCGG<br>ATAAAGTTGCAGGACCACTTCTGCGCTCGGCCCTTCCGGCTGGC<br>TGGTTTATTGCTGATAAATCTGGAGCCGGTGAGCGTGGGTCTCG<br>CGGTATCATTGCAGCACTGGGGCCAGATGGTAAGCCCTCCCGTA<br>TCGTAGTTATCTACACGACGGGGAGTCAGGCAACTATGGATGAA<br>CGAAATAGACAGATCGCTGAGATAGGTGCCTCACTGATTAAGCA<br>TTGGTAACTGTCAGACCAAGTTTACTCATATATACTTTAGATTG<br>ATTTACGCGCCCTGTAGCGGCGCATTAAGCGCGGCGGGTGTGGT<br>GGTTACGCGCAGCGTGACCGCTACACTTGCCAGCGCCCTAGCGC<br>CCGCTCCTTTCGCTTTCTTCCCTTCCTTTCTCGCCACGTTCGCC<br>GGCTTTCCCCGTCAAGCTCTAAATCGGGGGCTCCCTTTAGGGTT<br>CCGATTTAGTGCTTTACGGCACCTCGACCCCAAAAAACTTGATT

	TGGGTGATGGTTCACGTAGTGGGCCATCGCCCTGATAGACGGTT<br>TTTCGCCCTTTGACGTTGGAGTCCACGTTCTTTAATAGTGGACT<br>CTTGTTCCAAACTTGAACAACACTCAACCCTATCTCGGGCTATT<br>CTTTTGATTTATAAGGGATTTTGCCGATTTCGGCCTATTGGTTA<br>AAAAATGAGCTGATTTAACAAAAATTTAACGCGAATTTTAACAA<br>AATATTAACGTTTACAATTTAAAAGGATCTAGGTGAAGATCCTT<br>TTTGATAATCTCATGACCAAAATCCCTTAACGTGAGTTTTCGTT<br>CCACTGAGCGTCAGACCCCGTAGAAAAGATCAAAGGATCTTCTT<br>GAGATCCTTTTTTTCTGCGCGTAATCTGCTGCTTGCAAACAAAA<br>AAACCACCGCTACCAGCGGTGGTTTGTTTGCCGGATCAAGAGCT<br>ACCAACTCTTTTTCCGAAGGTAACTGGCTTCAGCAGAGCGCAGA<br>TACCAAATACTGTCCTTCTAGTGTAGCCGTAGTTAGGCCACCAC<br>TTCAAGAACTCTGTAGCACCGCCTACATACCTCGCTCTGCTAAT<br>CCTGTTACCAGTCAGGCATTTGAGAAGCACACGGTCACACTGCT<br>TCCGGTAGTCAATAAACCGGTAAACCAGCAATAGACATAAGCGG<br>CTATTTAACGACCCTGCCCTGAACCGACGACCGGGTCGAATTTG<br>CTTTCGAATTTCTGCCATTCATCCGCTTATTATCACTTATTCAG<br>GCGTAGCACCAGGCGTTTAAGGGCACCAATAACTGCCTTAAAAA<br>AATTACGCCCCGCCCTGCCACTCATCGCAGTACTGTTGTAATTC<br>ATTAAGCATTCTGCCGACATGGAAGCCATCACAGACGGCATGAT<br>GAACCTGAATCGCCAGCGGCATCAGCACCTTGTCGCCTTGCGTA<br>TAATATTTGCCCATGGTGAAAACGGGGGCGAAGAAGTTGTCCAT<br>ATTGGCCACGTTTAAATCAAAACTGGTGAAACTCACCCAGGGAT<br>TGGCTGAGACGAAAAACATATTCTCAATAAACCCTTTAGGGAAA<br>TAGGCCAGGTTTTCACCGTAACACGCCACATCTTGCGAATATAT<br>GTGTAGAAACTGCCGGAAATCGTCGTGGTATTCACTCCAGAGCG<br>ATGAAAACGTTTCAGTTTGCTCATGGAAAACGGTGTAACAAGGG<br>TGAACACTATCCCATATCACCAGCTCACCGTCTTTCATTGCCAT<br>ACGGAATTCCGGATGAGCATTCATCAGGCGGGCAAGAATGTGAA<br>TAAAGGCCGGATAAAACTTGTGCTTATTTTTCTTTACGGTCTTT<br>AAAAAGGCCGTAATATCCAGCTGAACGGTCTGGTTATAGGTACA<br>TTGAGCAACTGACTGAAATGCCTCAAAATGTTCTTTACGATGCC<br>ATTGGGATATATCAACGGTGGTATATCCAGTGATTTTTTTCTCC<br>ATTTTAGCTTCCTTAGCTCCTGAAAATCTCGATAACTCAAAAAA<br>TACGCCCGGTAGTGATCTTATTTCATTATGGTGAAAGTTGGAAC<br>CTCTTACGTGCCGATCAACGTCTCATTTTCGCCAAAAGTTGGCC<br>CAGGGCTTCCCGGTATCAACAGGGACACCAGGATTTATTTATTC<br>TGCGAAGTGATCTTCCGTCACAGGTATTTATTCGGCGCAAAGTG<br>CGTCGGGTGATGCTGCCAACTTACTGATTTAGTGTATGATGGTG<br>TTTTTGAGGTGCTCCAGTGGCTTCTGTTTCTATCAGCTGTCCCT<br>CCTGTTCAGCTACTGACGGGGTGGTGCGTAACGGCAAAAGCACC<br>GCCGGACATCAGCGCTAGCGGAGTGTATACTGGCTTACTATGTT<br>GGCACTGATGAGGGTGTCAGTGAAGTGCTTCATGTGGCAGGAGA<br>AAAAAGGCTGCACCGGTGCGTCAGCAGAATATGTGATACAGGAT<br>ATATTCCGCTTCCTCGCTCACTGACTCGCTACGCTCGGTCGTTC<br>GACTGCGGCGAGCGGAAATGGCTTACGAACGGGGCGGAGATTTC<br>CTGGAAGATGCCAGGAAGATACTTAACAGGGAAGTGAGAGGGCC<br>GCGGCAAAGCCGTTTTTCCATAGGCTCCGCCCCCCTGACAAGCA<br>TCACGAAATCTGACGCTCAAATCAGTGGTGGCGAAACCCGACAG<br>GACTATAAAGATACCAGGCGTTTCCCCCTGGCGGCTCCCTCGTG<br>CGCTCTCCTGTTCCTGCCTTTCGGTTTACCGGTGTCATTCCGCT<br>GTTATGGCCGCGTTTGTCTCATTCCACGCCTGACACTCAGTTCC

	GGGTAGGCAGTTCGCTCCAAGCTGGACTGTATGCACGAACCCCC<br>CGTTCAGTCCGACCGCTGCGCCTTATCCGGTAACTATCGTCTTG<br>AGTCCAACCCGGAAAGACATGCAAAAGCACCACTGGCAGCAGCC<br>ACTGGTAATTGATTTAGAGGAGTTAGTCTTGAAGTCATGCGCCG<br>GTTAAGGCTAAACTGAAAGGACAAGTTTTGGTGACTGCGCTCCT<br>CCAAGCCAGTTACCTCGGTTCAAAGAGTTGGTAGCTCAGAGAAC<br>CTTCGAAAAACCGCCCTGCAAGGCGGTTTTTTCGTTTTCAGAGC<br>AAGAGATTACGCGCAGACCAAAACGATCTCAAGAAGATCATCTT<br>ATTAATCAGATAAAATATTTGCTCATGAGCCCGAAGTGGCGAGC<br>CCGATCTTCCCCATCGGTGATGTCGGCGATATAGGCGCCAGCAA<br>CCGCACCTGTGGCGCCGGTGATGCCGGCCACGATGCGTCCGGCG<br>TAGAGGATCTGCTCATGTTTGACAGCTTATCATCGATGCATAAT<br>GTGCCTGTCAAATGGACGAAGCAGGGATTCTGCAAACCCTATGC<br>TACTCCGTCAAGCCGTCAATTGTCTGATTCGTTACCAATTATGA<br>CAACTTGACGGCTACATCATTCACTTTTTCTTCACAACCGGCAC<br>GGAACTCGCTCGGGCTGGCCCCGGTGCATTTTTTAAATACCCGC<br>GAGAAATAGAGTTGATCGTCAAAACCAACATTGCGACCGACGGT<br>GGCGATAGGCATCCGGGTGGTGCTCAAAAGCAGCTTCGCCTGGC<br>TGATACGTTGGTCCTCGCGCCAGCTTAAGACGCTAATCCCTAAC<br>TGCTGGCGGAAAAGATGTGACAGACGCGACGGCGACAAGCAAAC<br>ATGCTGTGCGACGCTGGCGATATCAAAATTGCTGTCTGCCAGGT<br>GATCGCTGATGTACTGACAAGCCTCGCGTACCCGATTATCCATC<br>GGTGGATGGAGCGACTCGTTAATCGCTTCCATGCGCCGCAGTAA<br>CAATTGCTCAAGCAGATTTATCGCCAGCAGCTCCGAATAGCGCC<br>CTTCCCCTTGCCCGGCGTTAATGATTTGCCCAAACAGGTCGCTG<br>AAATGCGGCTGGTGCGCTTCATCCGGGCGAAAGAACCCCGTATT<br>GGCAAATATTGACGGCCAGTTAAGCCATTCATGCCAGTAGGCGC<br>GCGGACGAAAGTAAACCCACTGGTGATACCATTCGCGAGCCTCC<br>GGATGACGACCGTAGTGATGAATCTCTCCTGGCGGGAACAGCAA<br>AATATCACCCGGTCGGCAAACAAATTCTCGTCCCTGATTTTTCA<br>CCACCCCCTGACCGCGAATGGTGAGATTGAGAATATAACCTTTC<br>ATTCCCAGCGGTCGGTCGATAAAAAAATCGAGATAACCGTTGGC<br>CTCAATCGGCGTTAAACCCGCCACCAGATGGGCATTAAACGAGT<br>ATCCCGGCAGCAGGGGATCATTTTGCGCTTCAGCCATACTTTTC<br>ATACTCCCGCCATTCAGAG

[a] — A lowercase “m” before a base indicates that the base was synthesized with a 2’-methoxy modification.

[b] — The underlined sequence corresponds to the intron sequence.

Table S2: Sequence identities to P.li.LSU.I2.

Intron	Sequence identity with P.li.LSU.I2
Arq.I1	60.27%
Arq.I2	57.91%
Arq.I3	58.42%
P.li.LSU.I1	56.69%

Table S3: Mfold predicted free energies of domains I, II, and III (D123) for different introns.

Intron	ΔG [kcal/mol]
Arq.I1	-211.29
Arq.I2	-245.49
Arq.I3	-223.45
P.li.LSU.I2	-156.79

Table S4: Parameters of kinetic model. Rates that were constrained to be equal have been highlighted.

T [°C]	Mg^{2+} [mM]	$k_{branching}$ [95% CI]	$k_{hydrolysis}$ [95% CI]	k_{break} [95% CI]	k_{conf} [95% CI]	%Pre-folded [95% CI]
37	20	0.137 (0.107, 0.177)	0.024 (0.018, 0.032)	0.00165 (0.00136, 0.00194)	0.0035 (0.0032, 0.0038)	8 (0.0, 16.0)
37	30	0.137 (0.107, 0.177)	0.024 (0.018, 0.032)	0.00165 (0.00136, 0.00194)	0.0035 (0.0032, 0.0038)	18.8 (16.4, 21.3)
37	40	0.137 (0.107, 0.177)	0.024 (0.018, 0.032)	0.00165 (0.00136, 0.00194)	0.0035 (0.0032, 0.0038)	24.3 (22.0, 26.7)
37	50	0.137 (0.107, 0.177)	0.024 (0.018, 0.032)	0.00165 (0.00136, 0.00194)	0.0035 (0.0032, 0.0038)	31.4 (28.8, 34.1)
50	20	0.086 (0.063, 0.116)	0	0.0061 (0.0042, 0.0084)	0.0043 (0.003, 0.0057)	10 (5.0, 14.0)
50	30	0.086 (0.063, 0.116)	0.011 (0.007, 0.016)	0.0016 (0.0006, 0.0026)	0.0043 (0.003, 0.0057)	67 (58.0, 77.0)
50	40	0.42 (0.35, 0.51)	0.23 (0.18, 0.29)	0.015 (0.011, 0.02)	0.016 (0.005, 0.028)	80 (75.0, 85.0)
50	50	0.8 (0.59, 1.1)	0.15 (0.11, 0.2)	0.006 (0.002, 0.011)	0.016 (0.005, 0.028)	74 (68.0, 79.0)
50	100	0.42 (0.35, 0.51)	0.04 (0.11, 0.2)	0.015 (0.011, 0.02)	0.016 (0.005, 0.028)	74 (68.0, 79.0)

Table S5: Full list of the tryptic sfGFP peptides measured, with their corresponding charge state, collision product, quantification trace and predicted retention time.

Peptide name	Charge state	Collision Product	Quantification trace	Predicted RT (min)
EDGNILGHK.3_1	3	1	328.359 > 529.225	3.5950
EDGNILGHK.3_2	3	2	328.359 > 454.277	3.6350
EDGNILGHK.3_3	3	3	328.359 > 341.193	3.6050
SAMPEGYVQER.3_1	3	1	423.137 > 531.289	5.8600
SAMPEGYVQER.3_2	3	2	423.137 > 432.22	5.8400
LEYNFNSHNVYITADK.4	4	1	483.026 > 547.309	8.3600
GEGEGDATNGK.2_1	2	1	518.007 > 662.31	1.4150
GEGEGDATNGK.2_2	2	2	518.007 > 605.289	1.4200

GEGEGDATNGK.2_3	2	3	518.007 > 545.184	1.4300
FEGDTLVNR.2_1	2	1	526.072 > 717.389	6.3350
FEGDTLVNR.2_1	2	2	526.072 > 602.362	6.3450
FEGDTLVNR.2_3	2	3	526.072 > 550.214	6.3500
SAMPEGYVQER.2_1	2	1	634.201 > 751.373	5.8300
SAMPEGYVQER.2_2	2	2	634.201 > 290.117	5.8450
LEYNFNSHNVYITADK.3_1	3	1	643.699 > 809.44	8.3700
LEYNFNSHNVYITADK.3_2	3	2	643.699 > 710.372	8.3400
DHMVLLEFVTAAGITHGMDELYK.4_1	4	1	648.753 > 985.481	15.0450
DHMVLLEFVTAAGITHGMDELYK.4_2	4	2	648.753 > 855.392	15.0600
DHMVLLEFVTAAGITHGMDELYK.4_3	4	3	648.753 > 838.413	15.0550
DHMVLLEFVTAAGITHGMDELYK.4_4	4	4	648.753 > 709.37	15.0500
LPVPWPTLVTTLTYGVQCFSR.3_1	3	1	793.942 > 796.377	10.4550
LPVPWPTLVTTLTYGVQCFSR.3_2	3	2	793.942 > 310.213	10.4250

Table S6: Measured retention times and integrated peaks of each tryptic sfGFP peptide of each sample.

Peptide name	Sample	Retention time	Area
EDGNILGHK.3_1	sfGFP Standard	3.595	5175
	ΔRNA	3.545	8
	2-pot	3.740	5456
	1-pot	3.745	374
EDGNILGHK.3_2	sfGFP Standard	3.595	42442
	ΔRNA	3.551	104
	2-pot	3.740	47730
	1-pot	3.745	3421
EDGNILGHK.3_3	sfGFP Standard	3.595	85717
	ΔRNA		
	2-pot	3.745	97875
	1-pot	3.745	6741
SAMPEGYVQER.3_1	sfGFP Standard	5.835	86641
	ΔRNA		
	2-pot	5.818	49722
	1-pot	5.835	2346
SAMPEGYVQER.3_2	sfGFP Standard	5.835	170670
	ΔRNA		
	2-pot	5.824	97493
	1-pot	5.840	4402
LEYNFNSHNVYITADK.4	sfGFP Standard	8.343	3684
	ΔRNA		
	2-pot	8.323	1084
	1-pot		
GEGEGDATNGK.2_1	sfGFP Standard	1.411	91288
	ΔRNA	1.372	24
	2-pot	2.072	33493
	1-pot	2.072	157
GEGEGDATNGK.2_2	sfGFP Standard	1.411	14000
	ΔRNA		
	2-pot	2.072	5197
	1-pot	2.072	22
GEGEGDATNGK.2_3	sfGFP Standard	1.411	3564
	ΔRNA	1.400	21

	2-pot	2.072	1788
	1-pot	2.072	10
FEGDTLVNR.2_1	sfGFP Standard	6.330	60641
	ΔRNA	6.341	24
	2-pot	6.314	100475
	1-pot	6.325	4394
FEGDTLVNR.2_2	sfGFP Standard	6.330	334244
	ΔRNA	6.358	26
	2-pot	6.314	579321
	1-pot	6.325	25199
FEGDTLVNR.2_3	sfGFP Standard	6.336	23331
	ΔRNA		
	2-pot	6.314	39014
	1-pot	6.325	1251
SAMPEGYVQER.2_1	sfGFP Standard	5.833	348004
	ΔRNA	5.817	52
	2-pot	5.822	313394
	1-pot	5.833	13600
SAMPEGYVQER.2_2	sfGFP Standard	5.839	241371
	ΔRNA		
	2-pot	5.822	205036
	1-pot	5.833	8597
LEYNFNSHNVYITADK.3_1	sfGFP Standard	8.345	5760
	ΔRNA	8.362	41
	2-pot	8.325	2715
	1-pot	8.342	254
LEYNFNSHNVYITADK.3_2	sfGFP Standard	8.345	23129
	ΔRNA	8.358	350
	2-pot	8.329	11108
	1-pot	8.339	1112
DHMVLLEFVTAAGITHGMDELYK.4_1	sfGFP Standard	15.029	34366
	ΔRNA	15.035	168
	2-pot	15.024	52028
	1-pot	15.012	15814
DHMVLLEFVTAAGITHGMDELYK.4_2	sfGFP Standard	15.029	17053
	ΔRNA	15.051	172
	2-pot	15.024	28182
	1-pot	15.018	8469

DHMVLLEFVTAAGITHGMDELYK.4_3	sfGFP Standard	15.029	61210
	ΔRNA		
	2-pot	15.024	94569
	1-pot	15.018	28941
DHMVLLEFVTAAGITHGMDELYK.4_4	sfGFP Standard	15.035	29731
	ΔRNA	15.024	1622
	2-pot	15.024	48370
	1-pot	15.018	16154
LPVPWPTLVTTLTYGVQCFSR.3_1	sfGFP Standard	10.398	5656
	ΔRNA		
	2-pot		
	1-pot		
LPVPWPTLVTTLTYGVQCFSR.3_2	sfGFP Standard	10.396	5803
	ΔRNA	10.523	3105
	2-pot	10.519	2933
	1-pot	10.523	3006

References

[1] S. Hoops, S. Sahle, R. Gauges, C. Lee, J. Pahle, N. Simus, M. Singhal, L. Xu, P. Mendes, U. Kummer, "COPASI--a COmplex PAthway SImulator", *Bioinformatics* **2006**, *22*, 3067.

[2] J. Schaber, "Easy parameter identifiability analysis with COPASI", *Biosystems* **2012**, *110*, 183.

[3] A. Doerr, D. Foschepoth, A. C. Forster, C. Danelon, "In vitro synthesis of 32 translation-factor proteins from a single template reveals impaired ribosomal processivity", *Sci Rep* **2021**, *11*, 1898.

[4] L. K. Pino, B. C. Searle, J. G. Bollinger, B. Nunn, B. MacLean, M. J. MacCoss, "The Skyline ecosystem: Informatics for quantitative mass spectrometry proteomics", *Mass Spectrom Rev* **2020**, *39*, 229.

[5] C. Zhang, Y. Zhang, A. M. Pyle, "rMSA: A Sequence Search and Alignment Algorithm to Improve RNA Structure Modeling", *J Mol Biol* **2023**, *435*, 167904.

[6] M. Costa, J. M. Fontaine, S. Loiseaux-de Goër, F. Michel, "A group II self-splicing intron from the brown alga Pylaiella littoralis is active at unusually low magnesium concentrations and forms populations of molecules with a uniform conformation", *J Mol Biol* **1997**, *274*, 353.

[7] J.-D. Pédelacq, S. Cabantous, T. Tran, T. C. Terwilliger, G. S. Waldo, "Engineering and characterization of a superfolder green fluorescent protein", *Nat Biotechnol* **2006**, *24*, 79.
